# Exploring Virtual Reality-Based Reminiscence Therapy on Cognitive and Emotional Well-Being in People with Cognitive Impairments: A Scoping Review

**DOI:** 10.3390/brainsci15050500

**Published:** 2025-05-13

**Authors:** Susanna Pardini, Riccardo Calcagno, Anna Genovese, Elio Salvadori, Oscar Mayora Ibarra

**Affiliations:** Digital Health Research, Centre for Digital Health and Well-Being, Fondazione Bruno Kessler, 38123 Trento, Italy; spardini@fbk.eu (S.P.); rcalcagno@fbk.eu (R.C.); agenovese@fbk.eu (A.G.); esalvadori@fbk.eu (E.S.)

**Keywords:** virtual reality, reminiscence therapy, cognitive impairments, emotional well-being, quality of life, engagement, customization, older adults, non-pharmacological therapy, scoping review

## Abstract

Background/Objectives: Virtual reality (VR) is increasingly being explored as a non-pharmacological therapy to enhance the well-being of people with cognitive impairment (PwCI). Studies suggest that VR-based interventions improve mood, reduce apathy, and enhance emotional engagement, making VR a valuable tool for cognitive and emotional support. This scoping review synthesizes evidence on VR-based reminiscence therapy (VRRT) for PwCI. It aims to map existing knowledge, highlight implementation challenges, and offer practical, technical design, and evidence-informed recommendations for clinical integration—building on prior reviews that have touched on these aspects, but placing a stronger and more structured emphasis on real-world applicability and translational insights. This review draws extensively on qualitative findings across the included studies to better capture contextual factors, user experiences, facilitator roles, and barriers to usability. Moreover, unlike previous research, we included only studies involving individuals—either directly or via proxies—with an age-related cognitive impairment, formally diagnosed by a qualified authority. Methods: A systematic search based on the PRISMA-ScR guideline identified 310 studies, of which 11 met the inclusion criteria. These studies assessed the effectiveness and feasibility of immersive VRRT. Research methodologies included longitudinal (*n* = 2), cross-sectional (*n* = 2), mixed-methods (*n* = 4), and randomized controlled trials (*n* = 3)—with most studies focusing on feasibility—with a cumulative sample size of approximately 287 participants. The quality of the included studies was generally moderate; common limitations included small sample sizes, short intervention periods, and limited control conditions. Results: The findings highlight VRRT’s potential to enhance engagement, emotional well-being, and cognitive function. However, usability challenges and technical limitations persist. While VR offers promising benefits, further research is needed to refine interventions, address personalization barriers, and assess long-term effects. Conclusions: This review underscores the importance of integrating VRRT into care programs and improving accessibility. Future research should enhance methodological rigor to ensure reliable outcomes and maximize VR’s impact on PwCI well-being. The scoping review protocol is registered a priori with the Center for Open Science (OSF) (registration type: OSF Preregistration, data registered: 15 November 2024, associated project: osf.io/r7jha, identifier: DOI 10.17605/OSF.IO/R7JHA).

## 1. Introduction

### 1.1. Virtual Reality for People with Cognitive Impairment (PwCI)

Virtual reality (VR) is a computer-generated immersive environment widely recognized for increasing medical training, rehabilitation, and therapy applications. VR has been used to simulate medical procedures for training healthcare professionals [1]. In rehabilitation, VR interventions have been applied to improve motor functions in patients recovering from strokes, traumatic brain injuries, and other physical impairments by creating interactive and engaging therapeutic exercises [2]. Additionally, VR is increasingly explored as a therapeutic tool for mental health conditions such as anxiety, PTSD, and dementia, demonstrating its ability to improve mood, reduce symptoms, and enhance quality of life [3,4]. Building on these diverse medical applications, VR is now being explored more specifically as a non-pharmacological therapy to support individuals with cognitive impairments. VR has also been explored as a tool to promote well-being in people with cognitive impairment (PwCI), serving as a non-pharmacological therapy to improve quality of life and well-being [5]. Even if the overall effectiveness remains difficult to assess—primarily due to significant methodological inconsistencies across studies—a scoping review indicates that VR-based interventions can be a safety activity that enhances mood, reduces apathy, and improves emotional engagement in people with cognitive impairment, making VR a valuable tool for addressing the complex needs of PwCI [6], especially for those with a dementia or mild cognitive impairment diagnosis. Another review underlined that while the use of VR shows exciting potential to meaningfully engage PwCI in residential aged care homes, there remain significant gaps in the research, especially regarding the ability of stakeholders to make well-informed decisions about the optimal timing, methods, and participants for introducing this technology. The key strength of using VR in residential aged care settings seems to be due to its ability to create opportunities for meaningful engagement, both socially and through purposeful activity [7].

### 1.2. Traditional Reminiscence Therapy and Its Challenges

As interest in supportive interventions for PwCI grows, reminiscence therapy (RT) has become one of the most widely used non-pharmacological approaches.

A longitudinal study [8] showed the potential of VR-based reminiscence therapy to enhance mood and preserve cognitive abilities during active intervention. This well-established psychosocial intervention involves revisiting past experiences through sensory stimuli such as photographs, music, or familiar objects. While RT has demonstrated modest positive effects in enhancing the quality of life, cognition, communication, and mood in PwCI patients, its overall efficacy is constrained by the limitations of traditional approaches, including a reduced opportunity to promote engagement, immersivity, and personalization [9,10]. Woods et al. [9] found that the effectiveness of RT is inconsistent and depends heavily on the context in which it is implemented. Care-home-based interventions seem to yield more positive outcomes, particularly for quality of life (QoL) and cognition, whereas community settings show fewer benefits. Moreover, Woods et al. [9] pointed out that further research is needed to refine RT approaches, particularly through high-quality randomized controlled trials. There is also potential for personalization, as tailoring RT to individual preferences and histories might enhance its impact.

### 1.3. Advancements Through VR-Based Reminiscence Therapy (VRRT)

To overcome the limitations of traditional RT, researchers have begun exploring digital platforms—including VR—as a means of delivering more immersive and personalized reminiscence experiences.

While traditional reminiscence therapy (RT) methods—such as the use of photographs, music, or storytelling—have demonstrated modest benefits in improving cognitive and emotional outcomes for people with cognitive impairment (PwCI) [9,10], these methods often lack the immersive, interactive, and highly personalized nature that modern digital tools can offer. Tablets, phones, and computers have been explored as platforms for digital reminiscence therapy, providing accessible and cost-effective alternatives [11]. However, these devices typically deliver experiences through flat screens, limiting sensory engagement, presence, and emotional resonance—factors crucial for individuals with cognitive decline [11,12].

Virtual reality (VR) distinguishes itself from other digital and traditional methods through its capacity to create immersive, three-dimensional environments that more closely mimic real-world experiences [5,7]. This heightened sense of presence and immersion plays a critical role in enhancing emotional engagement, stimulating autobiographical memory, and promoting cognitive activation. For older adults, particularly those with cognitive impairments, VR offers unique opportunities to foster deeper emotional connections, enhance memory recall, and increase engagement by simulating familiar places, events, and interactions [13,14]. Several studies have shown that VR-based reminiscence interventions lead to significantly greater improvements in clinical outcomes—such as mood enhancement, reduction in apathy, and improvements in verbal fluency, attention, and executive function—compared to traditional or non-immersive methods [12,13,14]. Immersive environments, particularly when personalized to users’ backgrounds and preferences, facilitate stronger emotional connections, deeper cognitive engagement, and more sustained therapeutic benefits. Therefore, VR’s capacity to provide immersive, emotionally resonant, and interactive experiences represents a substantial advancement over traditional RT approaches, offering a powerful tool to optimize clinical outcomes in dementia care and cognitive rehabilitation.

Despite these advantages, VR implementation in older populations faces important challenges. Usability issues, such as the complexity of navigation controls, the physical comfort of head-mounted displays, and the potential for motion sickness, need careful attention [15,16]. Nonetheless, studies have shown that with appropriately simplified interfaces, caregiver support, and careful content tailoring, VR experiences can be highly usable and acceptable for older adults [15,16,17].

The availability of VR technology is also improving. Standalone headsets are becoming more affordable and user-friendly, increasing their potential for adoption in care settings [13,15]. Content creation remains a significant hurdle, especially in producing personalized and culturally relevant VR experiences that align with the user’s personal history and preferences [14,18]. Developing such customized content requires considerable time, technical expertise, and financial resources [17,18]. However, advances in 360° photography, virtual-world-building tools, and participatory co-design methodologies with caregivers and users are opening up new avenues for scalable and meaningful VR interventions [19].

Consequently, VR offers an innovative and promising platform for reminiscence therapy by addressing the limitations of both traditional and flat-screen digital methods. This potential underscores the need to systematically review current VR-based reminiscence interventions, focusing on their feasibility, usability, emotional impact, and cognitive outcomes for PwCI.

### 1.4. Recent Research on Virtual Reality Reminiscence Therapy (VRRT)

Preliminary research suggests that immersive VR-based reminiscence therapy can yield immediate benefits, such as reduced anxiety and improved verbal fluency, particularly in elderly individuals within care facilities [12,13]. Beyond these short-term outcomes, VR has also shown promise in sustaining cognitive functions, preserving memory, and increasing attention in PwCI [14].

Recent systematic reviews have further enriched the field of VR-based reminiscence therapy (VRRT) for older adults. Lu et al. [20] conducted a scoping review analyzing 11 studies and highlighting the potential of fully immersive VR environments (FIVR) in enhancing psychological well-being, memory recall, and social engagement among older adults. They emphasized that virtual environments tailored to individuals’ personal histories—such as recreations of meaningful places and events—were effective in promoting emotional responses and cognitive stimulation. Both passive and interactive VR experiences demonstrated benefits, although interactive scenarios tended to elicit stronger engagement. Importantly, Lu et al. [20] noted that FIVR-based reminiscence interventions improved user engagement, reduced session fatigue, and were generally perceived as usable and acceptable, with only minor side effects such as occasional motion sickness or emotional distress. Nonetheless, evidence for improvements in anxiety, depression, apathy, and cognitive functions remained inconsistent across studies, largely due to small sample sizes, variability in intervention design, and a lack of rigorous long-term assessments. They recommended greater personalization of VR content, enhanced facilitator training, and the careful selection of stimuli to minimize adverse emotional reactions.

Ng et al. [21] performed a systematic review focusing on VRRT interventions across different settings, analyzing 22 studies. They classified VRRT systems into four core design aspects: context (personalized vs. generic environments), level of immersion, degree of interactivity, and mode of delivery. Personalized environments, where content was adapted to individuals’ backgrounds, memories, or preferences, were particularly effective in eliciting emotional and cognitive engagement. High immersion levels, achieved through head-mounted displays and multimodal sensory stimulation, significantly improved user experience and therapeutic outcomes. Interactivity varied widely, with some systems granting participants direct control and others relying on facilitator-guided navigation. Autonomy within the virtual environment was identified as a key factor in promoting emotional benefits, cognitive improvements, and overall satisfaction. Additionally, the supportive presence of a facilitator during sessions was considered essential for optimizing benefits and minimizing confusion.

Mao et al. [22] systematically reviewed VRRT interventions specifically for PwCI, including dementia and mild cognitive impairment (MCI). They found that VRRT could maintain cognitive functioning and reduce anxiety during active intervention periods. However, long-term improvements in quality of life, apathy, or depression remained inconclusive, mainly due to heterogeneity in study designs and variability in VR content. Challenges related to usability, particularly for individuals with more severe cognitive decline, were also reported, reinforcing the need for greater personalization and technological accessibility.

Similarly, Yang et al. [23] conducted a systematic review and meta-analysis focusing on VR interventions for older adults with MCI. Their results confirmed that VR-based cognitive training improved memory performance, attention, information processing speed, and executive function. Although effects on general cognitive function and physical health outcomes were modest, immersive VR systems and cognitive-focused interventions showed the greatest promise. Wang et al. emphasized the importance of integrating multisensory experiences and culturally relevant content to enhance efficacy and facilitate real-world implementation.

Collectively, these recent contributions highlight the dynamic and rapidly evolving nature of the VRRT field. They reinforce the importance of continuously updating research and practice with the latest evidence, paying particular attention to personalization, facilitator involvement, session structure, and technological accessibility. While recent reviews have deepened our understanding of therapeutic outcomes, they have also underscored the need for further investigation into user engagement, broader health outcomes, and the practical feasibility of real-world deployment.

Given the increasing interest in VRRT as an innovative approach to dementia care, the comprehensive exploration of these aspects is essential to bridge existing research gaps and develop optimized interventions. Continuous and systematic literature reviews are vital to monitor developments, document incremental advancements, and foster a nuanced and up-to-date understanding of the field. Our scoping review seeks to contribute to this effort by examining not only the broader landscape of VRRT applications, but also by placing a specific focus on feasibility, future advancements, and real-world implementation. Moreover, unlike previous research, we applied more stringent inclusion criteria to ensure a diagnostically homogeneous sample. We included only studies for which the direct recipients of treatments or, where proxies^†^ are used, the individuals they represent, have a diagnosed age-related cognitive impairment, mild or severe, assessed by an authority.

Additionally, while Mao et al.’s [22] review considered a wide range of participants, our study narrows its scope to older adults with mild to severe cognitive impairments, ensuring a targeted investigation into the challenges and opportunities relevant to this population.

In general, based on our current knowledge of the state of the art, while VR shows potential for RT, research on its application in reminiscence interventions remains limited. Studies have reported barriers to personalization in VR content, requiring significant time and financial resources to create individualized virtual experiences. Additionally, the long-term effects of VR-based RT interventions on patients with cognitive impairments still need to be explored. Nonetheless, personalization in VR scenarios is emerging as a critical area for enhancing user immersion and engagement, particularly for individuals with cognitive impairments, by creating environments aligned with user preferences.

### 1.5. Aims of the Current Scoping Review

The current scoping review aims to synthesize existing evidence on using VR-based RT for cognitive and emotional well-being in PwCI. By identifying research gaps and summarizing current findings, the review provides a foundation for developing innovative, feasible, and effective interventions to improve the well-being of PwCI with a diagnosis of dementia or mild cognitive impairment (MCI). In this scoping review, the initial focus on database searches for academic peer-reviewed literature ensures a rigorous and systematic examination of scientific studies related to the defined topic. Grounding the review in scientifically validated evidence provides a robust foundation for the subsequent exploration of broader practical implications and innovations found in gray literature. This phased approach strategically broadens the scope of analysis, integrating insights from theoretical foundations to practical applications within the field. Three key research questions guided the search strategy and data extraction process in the academic literature:

What are the critical factors influencing the successful integration of VRRT into care programs, including participant engagement, facilitator roles, cultural and demographic preferences, and innovations in interaction methods and customization?

How can methodological characteristics and feasibility outcomes inform the design of subsequent studies aiming for more rigorous objectives in highlighting data on effectiveness?

What type of VR content is most effective in maximizing the therapeutic benefits and emotional impact of virtual reality reminiscence therapy (VRRT) for individuals with neurocognitive disorders?

This methodology ensures a comprehensive understanding of the topic, facilitating the identification of established knowledge and areas requiring further investigation. To ensure terminological consistency and given that samples of the included studies primarily consisted of individuals with mild cognitive impairment (MCI) and dementia, we have chosen to use the more general term “patients with cognitive impairment” where appropriate. In this study, this umbrella term specifically refers only to individuals diagnosed with MCI or dementia [22].

## 2. Materials and Methods

### 2.1. Study Design

Two studies were undertaken as a structured approach to thoroughly map the existing evidence and summarize the scholarly literature on the specified topic [24,25]. The review adhered to a five-step process: (1) formulating the research question, (2) identifying pertinent studies, (3) selecting relevant studies, (4) organizing the information, and (5) synthesizing and presenting the outcomes [26]. The final search strategy was crafted through an iterative process to fine-tune database searches and enhance search terms. The methodology for this scoping review, encompassing study design and manuscript preparation, followed the PRISMA-ScR (Preferred Reporting Items for Systematic Reviews and Meta-Analyses Extension for Scoping Reviews) framework to ensure methodological precision and clarity [26].

### 2.2. Protocol and Registration 

The scoping review protocol is registered a priori with the Center for Open Science (OSF) (registration type: OSF Preregistration, data registered: 15 November 2024, associated project: osf.io/r7jha, identifier: DOI 10.17605/OSF.IO/R7JHA).

### 2.3. Eligibility Criteria

To systematically identify a comprehensive set of articles that contribute to addressing our research questions, we established a set of eligibility criteria to be applied iteratively through several rounds, as detailed in the paragraph about the selection procedure of sources. The criteria are divided into inclusion and exclusion categories and applied iteratively through several rounds to ensure a comprehensive and systematic selection of relevant studies. The criteria aim to minimize false negatives and false positives, ensuring that only studies meeting the defined standards are included in the review. The table provides a clear and structured overview of the criteria used to filter and select the studies, contributing to the rigor and validity of the scoping review process.

### 2.4. Information Sources and Search Strategy

**Figure 1:**
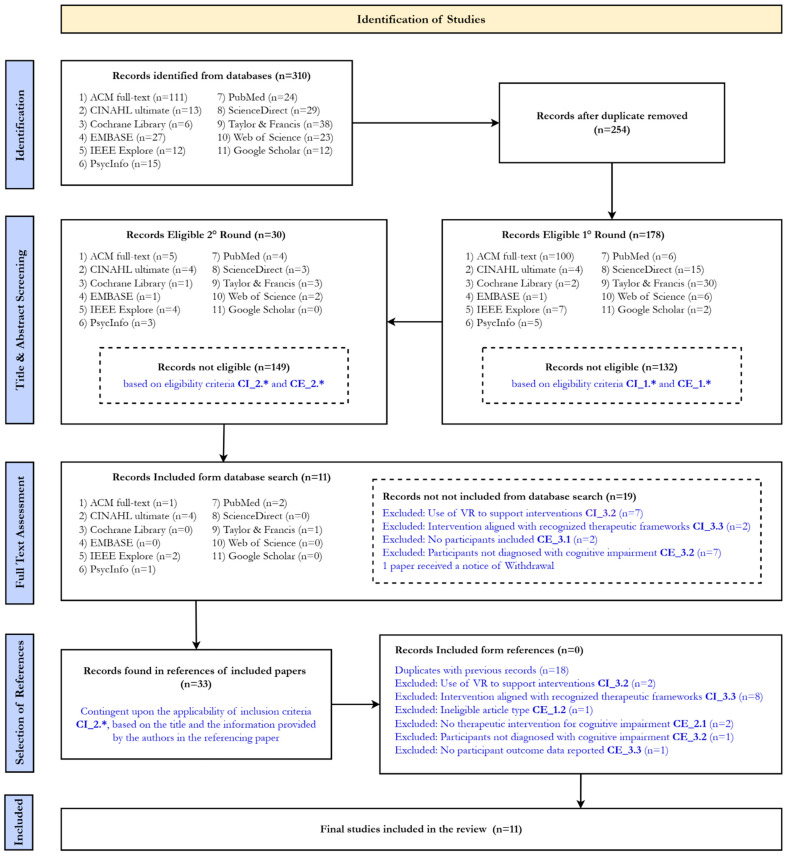
PRISMA flow diagram.

The research team conducted the scoping review search in October and November 2024 and focused only on published literature. The search included a range of databases, such as ACM, IEEE, PubMed, Web of Science, CINAHL, Cochrane, EMBASE, PsycINFO, PubMed, ScienceDirect, Taylor & Francis, and Google Scholar (Box 1) to identify academic, peer-reviewed journal articles and in proceeding papers. Reflecting the interdisciplinary scope of the study, the search encompassed databases from health sciences and engineering disciplines, ensuring a comprehensive review of relevant literature. The search strategy, including the keywords and search strings used, is summarized in Box 1. The research team supplemented the source identification process by including references cited within the full texts of publications included as a result of the database search. This approach was guided by the selection strategy detailed in the flowchart (Figure 1). Articles retrieved through the advanced search functions of all databases were imported into a shared reference collection using Mendeley Reference Manager [27] (Version 2.124.0), allowing all team members to identify and screen the references (1° round, 2° round). Subsequently, full-text reviews, the inclusion of references from included papers, data charting, and data extraction processes were leveraging on the collection of screened references exported in the software Sheets by Google [28].

Box 1 **Search strategy.**

- Databases: ACM, IEEE, PubMed, Web of Science, CINAHL, Cochrane, EMBASE, PsycINFO, PubMed, ScienceDirect, Taylor & Francis, and Google Scholar
- Limits and filters (where applicable):
  ○ Document type: Research article
  ○ Type of publication: Journals or in proceedings
  ○ Peer reviewed: True
  ○ Language: English
  ○ Excluding type of publication: Reviews and meeting abstract
- Date: All literature published up until October 2024
- Search string: #1 AND #2 AND #3 AND #4

#1: (((Neuro* OR Cognitive) AND (impairment* OR Decline OR Aging)) OR “aged care” OR “Amnestic Disorder” OR “Neurodegenerative Diseases” OR Dementia* OR Alzheimer* OR Parkinson*)

#2: (“Non-Pharmacological” OR Therapeutics OR therapy* OR intervention* OR treatment* OR rehabilitation OR Teletherapy OR Telemedicine OR Telehealth)

#3: (Reminiscence OR “Life Recall” OR “Biographical memo*” OR “Autobiographical memo*” OR “Life Narration” OR “nostalgic recount)

#4: (VR OR FIVR OR “Virtual Reality*” OR “Immersive virtual” OR “Immersive env*” OR “Augmented Reality” OR “Virtual Environment*” OR “Head-Mounted* Display*” OR headset* OR “Wearable Display*”).

### 2.5. Selection of Source of Evidence

The selection of sources of evidence was conducted in four sequential and distinct phases: 1st Round, 2nd Round, Inclusion Round, and Reference Inclusion Round. During the 1st Round (25–30 October 2024), one team member (RC) applied the eligibility criteria CI_1.* and CE_1.* (Table 1) to the records detected. This involved reviewing the publication webpages on journal websites and, when necessary, examining the abstracts. In the 2nd Round (30 October–5 November 2024), two team members independently screened titles and abstracts (SP, RC) based on the inclusion and exclusion criteria CI_2.* and CE_2.*. In the Inclusion Round (5–12 November 2024), three team members (SP, RC, ES) divided the sources of evidence equally to conduct a full-text review and evaluate their compliance with the eligibility criteria CI_3.* and CE_3.*. In the Reference Inclusion Round (12–13 November 2024), one member (RC) re-examined the texts included in the previous phase to extract a list of references. These references were assessed for inclusion based on the context of their citation and the title of the source, applying the CI_2.* criteria. Subsequently, the included texts underwent further review to ensure adherence to the remaining eligibility criteria outlined in Table 1. Discrepancies and uncertainties encountered during the 1st Round, 2nd Round, and Reference Inclusion Round were resolved through review by a team member who had not participated in the respective round. For the Inclusion Round, any discrepancies were resolved through team discussions and majority voting. As in Figure 1, eleven studies were included in the scoping review [8,13,15,16,17,18,19,29,30,31,32].

### 2.6. Data Charting Process

Three researchers (SP, RC, ES) collaboratively developed a data extraction template to outline the key information required for this review. Initially, each researcher independently populated the template with items commonly observed in previously published scoping reviews aligned with the conceptual framework of this study. Following this initial phase, the researchers reviewed the selected sources, populated empty template fields with data relevant to research objectives, and proposed additional items as necessary. They then compared their findings and, through consensus, removed items deemed non-informative for the text screening process. Any discrepancies or ambiguities were resolved through discussion, leading to iterative refinements of the template to ensure its accuracy and comprehensiveness.

### 2.7. Data Items

Data were extracted into an Excel worksheet with various categories that systematically captured information relevant to the studies’ objectives and research questions. The data, recorded either in narrative form or as nominal values, included the variables described in Table 2 and Table 3, and Table A1, Table A2 and Table A3 (Appendix A). 

### 2.8. Data Analysis

Quantitative data were analyzed using SPSS (Version 29.0.2.0 Armonk, NY, USA: IBM Corp.). Descriptive statistics (frequencies, means, standard deviations) were calculated on the following variables: database, country, aims of the studies, study design, sample size, gender, age, eligible criteria, type of diagnosis, diagnosis severity, psychological disorders in comorbidity, source of data, actors deployed during the experimental phases, type of interaction, facilitators during the experimental procedure, number of experimental sessions, minutes of exposure in the VR environment, comparison with control group, type of collection data (qualitative, quantitative), variable investigated, results, and features of the hardware and software equipment. We conducted a thematic analysis of the data derived from the results and conclusions of the studies included in the review, following Braun and Clarke’s [33] six-phase framework. First, in the familiarization phase, we (SP and AG) thoroughly read the methodology, results, discussion, and conclusion sections of each selected study. Second, during the initial coding phase, we systematically labeled and coded relevant segments across the dataset, identifying features pertinent to our research focus. Third, in the searching for themes phase (generation of themes), the coded data were organized into potential themes. SPSS software was utilized to assist in categorizing and examining the frequencies of the emerging thematic patterns, resulting in the identification of eight preliminary themes. In the reviewing themes phase, we refined and consolidated the themes through an iterative process, comparing, discussing, and evaluating the coherence and distinctiveness of the preliminary themes against the preliminary coded data. Fifth, in the defining and naming themes phase, we finalized the themes by clearly articulating the scope and content of each. Finally, in the report production phase, we described the thematic findings in terms of frequency distributions. Throughout the analysis, we applied an inductive, data-driven approach, allowing the themes to emerge organically from the data rather than being informed by pre-existing theoretical frameworks [33].

## 3. Results

### 3.1. Overview 

Eleven studies were included in this scoping review, selected from an initial pool of 310 studies identified through database searches. We organized the primary information from the 11 included documents into Table 2 and Table 3, and Table A1, Table A2 and Table A3 (Appendix A) summarize the main variables analyzed for each study. Figure 1 outlines the process based on PRISMA (Preferred Reporting Items for Systematic Reviews and Meta-Analyses) guidelines. In the following sections, we will present the results of the analysis. More detail about data are in the Supplementary Materials section.

### 3.2. Characteristics of the Study

#### 3.2.1. Databases and Geographic Distribution 

**Figure 2:**
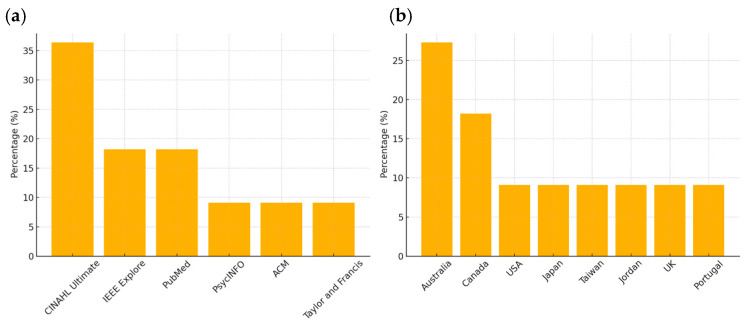
Distribution of sources and geographic origin of included studies. (**a**) Proportion of included studies retrieved from each bibliographic database. (**b**) Geographic distribution of the included studies, based on the country of the primary author’s affiliation. Percentages indicate the relative contribution to the total study pool.

Most studies were sourced from CINAHL Ultimate (36.4%), with smaller proportions from IEEE Explore and PubMed (18.2% each). Studies originated primarily from Australia (27.3%) and Canada (18.2%), with single contributions from the USA, Japan, Taiwan, Jordan, the UK, and Portugal (Table 2). In Figure 2 are the frequencies of studies sourced from each database and geographic distribution.

#### 3.2.2. Study Objectives and Study Designs 

Most studies (36.4%) evaluated both the feasibility and effectiveness of immersive VR reminiscence therapy (I-VRRT). Others focused on effectiveness alone (18.2%), co-design processes (18.2%), or comparative analysis with non-immersive or traditional therapies (27.3%). The design types varied, with exploratory/feasibility/mixed methods dominating (36.4%), and a minority adopting longitudinal, RCT, or hybrid formats. More than half were multi-center studies, and 81.8% lacked a control group (Table A1, Appendix A).

**Figure 3:**
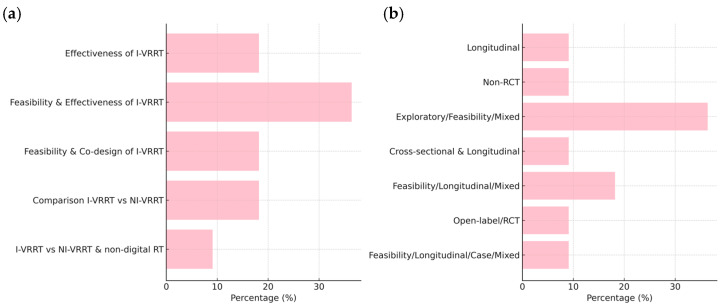
Overview of study objectives and research designs. (**a**) Frequency of primary research objectives across studies, including effectiveness evaluation, feasibility assessment, co-design processes, and comparative analyses. (**b**) Distribution of study designs, highlighting exploratory/mixed methods dominance, and the use of longitudinal, non-randomized, and randomized trial formats.

Figure 3 presents the frequency distribution of studies regarding the study objectives and study designs.

#### 3.2.3. Participant Characteristics

Neurocognitive disorder diagnosis (81.8%) was the most used inclusion criterion, followed by comorbid psychopathological conditions (54.5%). Diagnostic variety was broad, with most participants classified under major neurocognitive disorder (45.5%).

**Figure 4:**
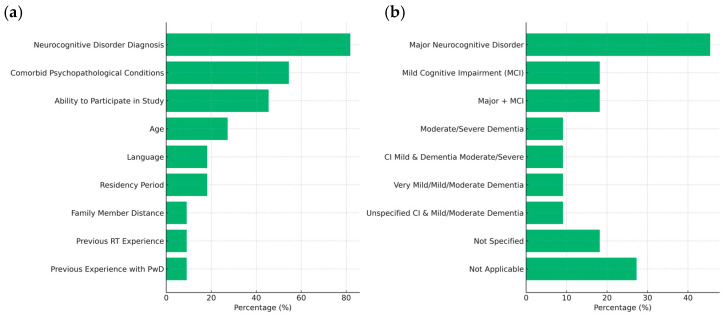
Distribution of participant inclusion criteria and cognitive diagnoses. (**a**) Frequency of inclusion criteria reported across selected studies (e.g., neurocognitive diagnosis, comorbidities, age). (**b**) Distribution of reported cognitive diagnoses among participants, including major neurocognitive disorder, mild cognitive impairment (MCI), and mixed or unspecified conditions. Percentages reflect the proportion of studies reporting each item.

The participants were mostly inpatients (90.9%), with an average age of 81.7 years. Females were more frequently represented, and the average sample size was 23 participants. The roles of caregivers, staff, researchers, and family members varied, often in complex or combined configurations (Table A1, Appendix A). Figure 4 illustrates the frequency distribution of the participants’ characteristics.

#### 3.2.4. Data Collection Measures 

**Figure 5:**
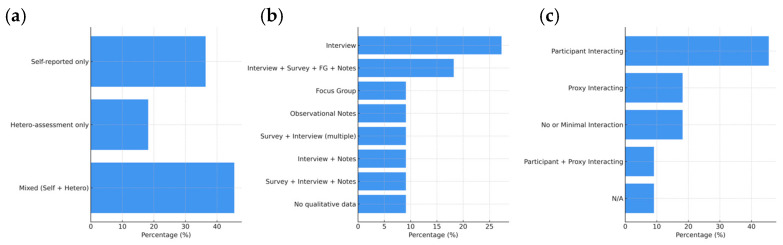
Overview of data collection methods and user interaction types. (**a**) Distribution of assessment modalities used in the studies, including self-reported, hetero-assessment, and mixed methods. (**b**) Qualitative data collection techniques reported, such as interviews, focus groups (FGs), surveys, and observational notes. (**c**) Categorization of user interaction levels within VR settings, distinguishing between participant-only, proxy-only, combined interaction, or minimal engagement. Percentages indicate the frequency of use across studies.

Data were primarily collected using mixed assessment methods (45.5%), combining self-reported and hetero-assessment tools. The most frequent qualitative method was interviews (27.3%), and quantitative tools included cognitive assessments and demographic profiling. Regarding interaction, participants were actively engaged in 45.5% of studies, while others used proxy-based or minimal interaction setups. In most studies (54.5%), facilitators discussed RT materials with participants (Table A2, Appendix A). Figure 5 displays the frequencies related to data collection measures.

#### 3.2.5. Hardware and Software Components

**Figure 6:**
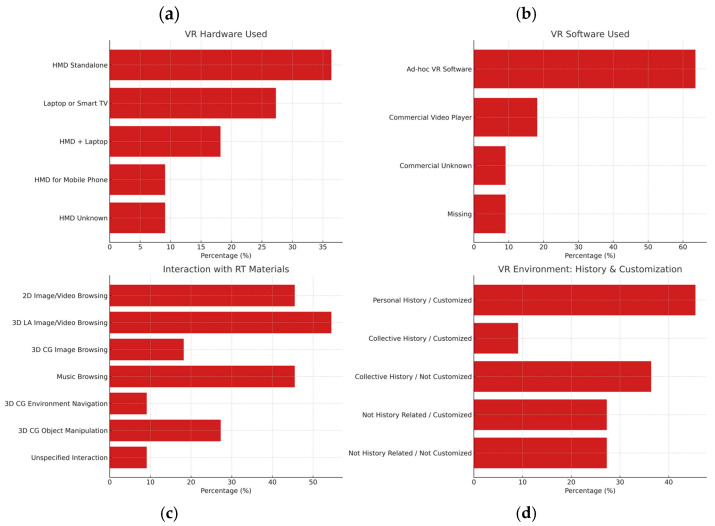
Distribution of hardware, software, interaction modalities, and environment features in VR-based reminiscence therapy. (**a**) Types of hardware used in the included studies, including standalone head-mounted displays (HMDs), laptops, mobile devices, and combinations thereof. (**b**) VR software employed, distinguishing between custom-built (“ad hoc”) platforms and commercial or unspecified tools. (**c**) Modes of user interaction with reminiscence therapy (RT) content, including 2D/3D browsing, manipulation of virtual objects, and audio material engagement. (**d**) Categorization of VR environments based on historical relevance (personal, collective, or unrelated to history) and the degree of customization involving user input.

The most commonly used hardware was standalone HMDs (36.4%), often paired with custom-built VR software (63.6%). Three-dimensional live-action image/video browsing (54.5%) and music track navigation (45.5%) were common interaction modalities. Some studies allowed manipulation or navigation within 3D environments. The most frequent VR setup was “personal history/customized” (45.5%), though other combinations of collective or non-history-based environments were also used (Table A3, Appendix A). Figure 6 presents the frequency of hardware and software component use.

### 3.3. Thematic Analysis: Key Results 

The thematic analysis presented in this paper was extrapolated from the independent work of two blinded authors. These authors systematically identified key themes and subthemes related to the results and discussion sections of the paper included. While the frequency of themes is reported for transparency, we acknowledge that percentages derived from a small sample size (*n* = 11) have limited statistical power. Therefore, rather than focusing on quantitative comparisons, we place stronger emphasis on identifying major thematic patterns, qualitative trends, and the relationships between themes. The analysis uncovered diverse insights into the application of immersive and non-immersive VR in therapeutic and research settings, particularly for older adults and individuals with cognitive impairments. The identified themes encompass key aspects such as participant engagement, emotional impact, usability, and cognitive outcomes. Each theme is supported by specific quotes from the literature, offering a comprehensive understanding of the benefits, challenges, and recommendations for VR-based interventions. 

More information about the results extrapolated from the selected papers is provided in Table A4, Table A5, Table A6, Table A7, Table A8, Table A9, Table A10 and Table A11 (Appendix B) and in the following sub-paragraphs.

#### 3.3.1. Theme 1: Participation and Engagement

High participation rates in VR-based studies and interventions were consistently reported (*n* = 10, 90.9%). Factors contributing to this aspect especially include the engaging and immersive nature of VR and the ability to customize experiences. These findings highlight VR’s potential as a tool for enhancing participation in therapeutic and recreational activities (*High Participation Rate subtheme*). VR fosters sustained engagement across multiple sessions. Enhanced conversational, behavioral, and kinetic engagement was consistently reported, particularly in structured sessions like RT. This highlights VR’s potential to maintain user interest and participation over time, even among populations with cognitive impairments (*Sustained Engagement theme*). The data were recorded in nine studies (81.8%). The documents collectively illustrate that VR is more effective in fostering emotional, conversational, and behavioral engagement compared to flat screens (NI-VRRT). This is attributed to the immersive and interactive features of VR, which provide a heightened sense of presence and involvement. Participants’ preference for I-VRRT over NI-VRRT is a recurring finding, reinforcing I-VR’s potential in therapeutic and recreational settings (*I-VRRT* vs. *NI-VRRT subtheme*). The data were recorded in three studies (27.3%). The papers consistently highlight the impact of immersive VR experiences on active participation and engagement. Factors such as tailored content, high visual realism, and a sense of presence significantly contribute to users’ involvement. The immersive nature of VR proves particularly effective in therapy settings, enabling participants to actively engage and maintain focus over multiple sessions (*Immersion and Active Participation theme*; the data were recorded in six studies (54.5%). The documents illustrate how VR fosters an enhanced sense of presence through immersive and realistic environments. Key factors include spatial and social presence, copresence, and active engagement in tailored activities. Indeed, participants’ positive feedback regarding their sense of immersion highlights the extent to which they felt engaged in the virtual world while remaining aware of their caregivers outside of it. Additionally, the theme of “being alone” emerged multiple times, suggesting that participants found solitude in the virtual environment to be a positive and enjoyable experience [15]. Another experience participants had during the VR experience related to social presence—the personal perception of being with a “real” individual in a virtual context. These immersive qualities also amplify the therapeutic impact of VR in settings like reminiscence therapy (*Enhanced Sense of Presence theme*; the data were recorded in five studies (45.5%). To summarize, VR sessions demonstrated strong adherence and low attrition rates, highlighting their acceptability and practicality in therapeutic contexts. A significant majority of studies reported elevated levels of engagement, emphasizing VR’s feasibility for integration into care programs. Simplified controls and interfaces were recommended to enhance accessibility. Participants showed consistent involvement over time, indicating VR’s potential for long-term use and meaningful therapeutic outcomes. The immersive nature of VR fosters more active and focused participation compared to flat-screen experiences by creating a heightened sense of presence. In Table A4 (Appendix B), all of the subthemes that emerged from the thematic analysis are described in detail. To summarize, rather than emphasizing numerical frequency alone, the findings across studies point to strong and recurring qualitative patterns: immersive VR environments consistently fostered active engagement, emotional involvement, and sustained participation. Key factors included realism, personalized or meaningful content, and a heightened sense of presence. These elements—alongside simplified controls and facilitator support—emerged as central to VRRT’s therapeutic potential. Importantly, users’ positive feedback across sessions highlights that engagement in VRRT is not merely novelty-driven, but potentially sustainable over time, reinforcing its value for integration into long-term care settings.

#### 3.3.2. Theme 2: Usability and Feasibility 

The thematic analysis outcomes revealed how VR interventions are not only feasible, but also hold potential for integration into care programs for older adults and individuals with dementia. These findings suggest that VR could significantly enhance non-pharmacological care strategies in institutional and community settings (VR is Feasible to be Integrated in Care Program subtheme; *n* = 10, 90.9%). The documents underline VR’s user-friendly nature, with high ease-of-use scores and positive feedback from both participants and caregivers. Minor technical issues were quickly addressed, and guided instructions further enhanced user experiences (Ease of Use subtheme; *n* = 11, 100%). User-friendly interfaces in VR systems and web-based tools are pivotal in ensuring accessibility and satisfaction. Features such as customizable layouts, intuitive navigation, adjustable hardware, and support for training significantly enhance the usability and adoption of these technologies in therapeutic and care settings (User-Friendly Interface subtheme; *n* = 4, 36.4%). Navigation and controls in VR systems are highly satisfactory for users, with features like hand controls, adjustable hardware, and intuitive interfaces enhancing the user experience (High Satisfaction with Navigation and Controls subtheme; *n* = 4, 36.4%). The data underscore the importance of simplifying VR controls to improve accessibility and usability for diverse user groups, including older adults and individuals with dementia. Key suggestions include reducing controller complexity, integrating alternative interaction methods like hand-tracking, and providing user-friendly interfaces (Simplifying Controls to Enhance Accessibility and Usability subtheme; *n* = 3, 27.3%). The documents collectively emphasize that VR interventions are well tolerated across diverse user groups, including older adults and individuals with dementia. Minor side effects, such as slight discomfort or eyestrain, were occasionally reported but did not detract from participants’ overall positive experiences and willingness to re-engage (Overall Well-tolerated Tool subtheme; *n* = 4, 36.4%). The data highlight the necessity of assistance for navigating web apps, particularly for elderly users or those with cognitive impairments. Key suggestions include providing training videos, guided instructions, and technical support. Assistance from caregivers or family members significantly enhances accessibility and usability, underscoring the role of support systems in ensuring the effective adoption of such technologies (Assistance Required for Web App Navigation subtheme; *n* = 3, 27.3%). The data highlighted the adaptability of VR interventions to meet the diverse needs of residents in aged care settings. Key factors include the customization of flexible session structures, and accommodations for cognitive and physical challenges (Adaptability to Resident Needs subtheme; *n* = 4, 36.4%). Caregivers demonstrated strong acceptance of VR-based interventions, citing their benefits in enhancing engagement, streamlining therapy processes, and improving caregiver–resident interactions. However, they also emphasized the importance of training, technical support, and further customization to ensure seamless adoption (Caregiver Acceptance subtheme; *n* = 1, 9.1%). In general, across the reviewed studies, usability and feasibility were consistently described as key enablers for the implementation of VRRT in clinical and caregiving contexts. Rather than focusing on isolated percentages, a broader pattern emerges: participants and caregivers generally found VR systems accessible and well tolerated when adequate support structures, such as facilitator guidance and simplified interfaces, were in place. Qualitative feedback emphasized the importance of intuitive navigation, ergonomic equipment, and flexible session design tailored to individual cognitive and physical needs. These insights underscore VRRT’s practicality and suggest that, with proper adaptation, it can be seamlessly integrated into care programs for PwCI. The detailed thematic analysis presented in Table A5 (Appendix B) elaborates on these emergent subthemes and their implications. 

#### 3.3.3. Theme 3: Emotional and Interpersonal Relationship Impact 

The data highlighted the positive emotional impacts of VR therapy, particularly in reducing anxiety and depression while promoting joy and relaxation. Key drivers include the recall of positive memories, enhanced emotional connections, and overall engagement in immersive environments (Positive Emotional Impact subtheme; *n* = 10, 90.9%). A positive effect of VR and reminiscence therapy in restoring mood and anxiety and enhancing emotional well-being emerged. Factors such as immersive environments and personalized content contribute significantly to this outcome (Mood Restoration subtheme; Reduction in Anxiety Symptoms subtheme; *n* = 4, 36.4%). VR-based interventions significantly reduce apathy levels, as evidenced by both quantitative measures and observable behavioral changes (Reduction in Apathy Score subtheme; *n* = 4, 36.4%). In general, users found VR sessions to be enjoyable and engaging (Increased Enjoyment and Likeability subtheme; *n* = 4, 36.4%). Moreover, the potential of VR emerged to simulate and promote social interactions, and create shared experiences, even in cases of geographical separation or physical distancing (Enhanced Social Presence and Co-presence subtheme; *n* = 5, 45.5%). These technologies not only improve communication, but also provide shared joyful experiences, enhancing the overall caregiving process and emotional well-being of both parties (Strengthened Resident–Caregiver Relationships subtheme; *n* = 5, 45.5%). The findings underscore the therapeutic potential of leveraging nostalgia and emotional engagement for mental health and quality of life improvements among older adults and individuals with dementia (Emotional Connection and Nostalgia subtheme; *n* = 5, 45.5%). Finally, reminiscence therapy, particularly in VR settings, has a role in restoring and maintaining a sense of self-worth and personal identity (Sense of Identity and Personal Value theme; *n* = 4, 36.4%). The results show that the qualitative evidence across the reviewed studies strongly supports VRRT’s capacity to evoke positive emotional responses and strengthen interpersonal connections. While individual outcome measures varied, a consistent trend emerged: participants experienced enhanced mood, reduced anxiety and apathy, and increased enjoyment during VR sessions. Particularly meaningful were the emotional effects linked to personalized or nostalgic content, which fostered a sense of identity and belonging. Additionally, VR was shown to support shared experiences and social presence, enhancing caregiver–resident relationships. These findings reinforce the therapeutic potential of VRRT not only as a cognitive tool, but also as an emotionally enriching and socially supportive intervention (Table A6, Appendix B). 

#### 3.3.4. Theme 4: Cognitive Function Outcomes 

Table A7 (Appendix B) focuses on the cognitive function outcomes associated with VR interventions, emphasizing their potential to improve cognitive abilities while also addressing the mixed results reported in the literature. One of the key findings is the significant cognitive improvement observed in some studies, particularly in areas like memory recall (*n* = 3, 27.3%) and overall cognitive functioning (*n* = 2, 18.2%). For example, tailored VR experiences such as personalized reminiscence sessions were shown to enhance memory recall and promote cognitive engagement, especially among individuals with mild cognitive impairment or dementia. 

However, in some papers, no significant cognitive improvements were detected (*n* = 3, 27.3%). These findings highlight the variability in VR’s effectiveness, suggesting that while technology may engage users, it does not always result in measurable changes in cognitive outcomes. This points to the need for further refinement of VR interventions and more robust experimental designs to better assess their cognitive impact. The findings reveal the dual nature of cognitive outcomes in VR research, with evidence supporting both its potential benefits and its limitations. The mixed results underline the importance of tailoring interventions to specific populations, incorporating personalized content, and adopting rigorous methodologies to maximize VR’s cognitive benefits. The reviewed studies present a mixed picture regarding cognitive outcomes, with some reporting improvements in memory and general cognitive functioning and others observing no measurable changes. However, qualitative findings offer valuable nuance: participants often showed signs of increased cognitive engagement during VRRT sessions, particularly when content was personalized and emotionally resonant. Rather than offering definitive proof of cognitive enhancement, these studies highlight the potential of VRRT to support cognitive stimulation through immersive and meaningful experiences. This underscores the importance of tailoring interventions to user profiles and calls for more rigorous designs to clarify VRRT’s long-term cognitive effects.

#### 3.3.5. Theme 5: Challenges

Table A8 (Appendix B) details the findings focusing on challenges associated with VR interventions. Moreover, various barriers and limitations that impact the effectiveness and usability of VR in therapeutic and recreational settings are revealed. Even though Theme 2 revealed that, in some cases, navigation and controls in VR systems are highly satisfactory for users, and minor side effects, such as slight discomfort or eyestrain, were occasionally reported, one of the prominent challenges that emerges is physical comfort. This highlights the fact that participants often experience discomfort with VR devices, particularly head-mounted displays (HMDs), due to improper fit, excessive weight, or limited adjustability. Another recurring issue is the complexity of navigation, as older users sometimes struggle with remembering functions and coordinating hand movements using controllers, underscoring the need for simplified and intuitive interfaces. Physical motion constraints also emerge as a critical barrier, with participants reporting difficulties navigating fully immersive environments, particularly if they have limited mobility or are prone to motion sickness. Another challenge involves limited interpersonal interaction, where the use of VR headsets can reduce face-to-face engagement between participants and caregivers, potentially impacting social connection and interaction quality. Indeed, while two studies [24,29] highlighted positive feedback on sense of immersion, emphasizing how engaged participants felt in the virtual world while remaining aware of their caregivers outside of it, another study found that the use of an HMD can sometimes hinder interaction between participants and researchers or therapists [29]. To enhance quality-of-life outcomes, fostering a more direct interpersonal connection may be necessary in certain cases. Therefore, it is crucial to consider the characteristics of the setting and individual interpersonal differences. The evidence of different degrees of technological familiarity among users significantly influence their ability to adapt to VR environments. This variability suggests a need for training and accessible guidance to support participants with little or no prior exposure to VR technology. Additionally, the analysis notes variability in participant engagement, with some individuals showing less interest or tolerance for extended VR sessions, necessitating flexibility in session lengths. To summarize, the thematic analysis emphasizes the importance of addressing these challenges to maximize the potential of VR interventions. Recommendations include simplifying controls, providing external guidance, ensuring ergonomic designs, and tailoring VR experiences to meet the unique needs and preferences of older adults and cognitively impaired individuals. The themes captured in Table A5 underscore the necessity of overcoming these obstacles to make VR more effective, accessible, and user-friendly in diverse care contexts. Here, the main extrapolated subthemes are summarized: (i) *Physical Comfort of Equipment* (*n* = 9, 81.8%); (ii) *Navigation Complexity for Older Users* (*n* = 8, 72.7%); (iii) *Physical Motion as a Critical Barrier* (*n* = 7, 63.6%); (iv) *Interactive Features in VR* (*n* = 2, 18.2%); (v) *Limited Interpersonal Interaction* (*n* = 1, 9.1%); (vi) *Different Levels of Familiarity with Technology that Impact on the Ability to Adapt to VR Environment* (*n* = 4, 36.4%); and (vii) *Participant Engagement Variability* (*n* = 4, 36.4%). To summarize the current results, it is important to point out that, while VRRT demonstrates promise, several recurring challenges were noted across studies, offering a clearer picture of barriers to real-world implementation. Discomfort with head-mounted displays, navigation complexity, motion sensitivity, and limited technological familiarity were common concerns, particularly among older adults with cognitive decline. Importantly, these issues often intersected with reduced engagement or reliance on caregiver assistance. Thematic analysis revealed that addressing these barriers requires user-centered design, simplified interaction mechanisms, and adaptable session formats. The consistency of these concerns across studies signals a critical area for refinement to ensure that VRRT is both accessible and effective across diverse care environments.

#### 3.3.6. Theme 6: Expressed Preferences Related to VR Scenarios

Table A9 (Appendix B) describes the preferences related to VR scenarios, emphasizing the importance of aligning VR content with user interests and therapeutic objectives. A recurring theme is the enjoyment derived from nature-based VR content, such as serene outdoor landscapes and calming environmental scenes, which create a relaxing and engaging atmosphere for participants. The incorporation of realistic elements in VR experiences is also highlighted as critical for enhancing memory recall and immersion. Participants often express greater emotional and cognitive connection when the VR environment closely resembles real-life settings. In Table A9 a strong preference for reminiscence-oriented content is also noted, including familiar or meaningful locations that evoke personal memories and foster a sense of security and nostalgia. This type of content is particularly effective in reminiscence therapy. While realism and familiarity are valued, the table emphasizes the need for variety in VR content to cater to diverse user preferences, ensuring that scenarios remain engaging and tailored to individual needs. Themes of relaxation also play a key role, as relaxing VR environments improve user performance and create a positive emotional state conducive to therapy. Finally, the analysis reveals a preference for I-VRRT experiences over NI-VRRT, with participants indicating a stronger sense of presence and enjoyment in immersive settings. Flexibility in the administration of VR sessions, such as customizing session duration and content delivery to suit individual preferences, is also highlighted as essential for optimizing user engagement and therapeutic outcomes. The preferences captured underscore the importance of designing VR experiences that are not only immersive and engaging, but also adaptable and responsive to the unique needs of each participant. In general, participants expressed strong preferences for realistic, meaningful, and nature-based content. Immersive VR experiences were favored over flat-screen options. Customized reminiscence-oriented and nature-based scenarios significantly enhanced engagement and therapeutic outcomes. Moreover, tailored session lengths, interaction methods, and content improved the accessibility and effectiveness of VR interventions for diverse user needs. Here, the main extrapolated subthemes are summarized: (i) Enjoyment for Nature-Based VR Content (*n* = 2, 18.2%); (ii) Realistic Elements (*n* = 7, 63.6%); (iii) Enjoyment of Reminiscence-Oriented Content (like familiar or meaningful locations) (*n* = 11, 100%); (iv) Variety in Content (*n* = 2, 18.2%); (v) Relaxing Themes (*n* = 9, 81.8%); (vi) Preferences for I-VRRT vs. NI-VRRT (*n* = 1, 9.1%); and (vii) Flexibility in VR Administration (*n* = 6, 54.5%).

Participants across multiple studies expressed clear preferences for VR content that was emotionally meaningful, realistic, and calming. Nature-based environments, familiar locations, and reminiscence-oriented scenarios consistently fostered greater immersion and emotional engagement. Qualitative feedback emphasized that personalization—even when subtle—enhanced the therapeutic value of sessions by evoking nostalgia and reinforcing identity. Additionally, preferences for immersive VR over non-immersive formats were linked to a stronger sense of presence and enjoyment. Flexibility in session design, including customizable durations and content, was also seen as essential for accommodating diverse cognitive and emotional needs. These preferences underscore the importance of tailoring VRRT experiences to individual users.

#### 3.3.7. Theme 7: Technical Design Recommendations

In Table A10 (Appendix B), there is information about technical design recommendations for improving VR experiences, focusing on enhancing usability, accessibility, and overall user satisfaction. A key theme is the importance of refining visual and auditory feedback, such as improving resolution, graphics quality, and soundscapes, to create more engaging and immersive environments. Enhanced realism is further emphasized, with recommendations to optimize object modeling, lighting, and camera rendering to ensure lifelike visuals and greater user immersion. In Table A10, the need for alternative interaction methods is also highlighted, particularly for users who face challenges with standard touch-based or motion-based controls. Suggestions include tangible interfaces, voice commands, and simplified interaction devices to improve accessibility for older adults or those with physical or cognitive impairments. Additionally, the integration of personalized reminiscence content, such as user-specific photos, familiar settings, or meaningful imagery, has been identified to foster deeper emotional connections and enhance therapeutic outcomes. Another focus is on increasing interactivity within VR environments, allowing users to engage actively with objects and scenarios, which enhances immersion and makes the experience more meaningful. Simplifying navigation systems is also a recurring recommendation, ensuring that VR controls are intuitive and user-friendly to reduce frustration and confusion. Table A7 underscores the importance of addressing cybersickness symptoms, advocating for design strategies like optimizing frame rates and minimizing disorienting movements to reduce discomfort. Facilitator assistance during sessions is recommended to provide guidance and support, especially for first-time users or those with limited technical familiarity. Flexibility in scene selection is also encouraged, allowing users to choose or customize scenarios to suit their preferences and therapeutic needs. Lastly, physical comfort is addressed with suggestions for improving the fit and ergonomics of headsets, such as adjustable straps and lighter materials, to ensure extended use without discomfort. The recommendations aim to address technical challenges and optimize VR design for therapeutic and recreational applications, particularly for older adults and individuals with specific needs. Here, the main extrapolated subthemes are summarized: (i) Adjustments for Better Visual and Audio Feedback (*n* = 10, 90.9%); (ii) Improved Object and Camera Rendering for Realism (*n* = 4, 36.4%); (iii) Tangible Interfaces for Non-Touch-Based Users (*n* = 4, 36.4%); (iv) Incorporation of Personalized Reminiscence Content (*n* = 5, 45.5%); (v) Enhanced Interactivity (*n* = 4, 36.4%); (vi) Improving/Simplifying Navigation (*n* = 3, 27.3%); (vii) Minimizing Cybersickness Symptoms (*n* = 2, 18.2%); (viii) Facilitator Assistance: Provide trained staff to guide participants during sessions for a smoother experience (*n* = 5, 45.5%); (ix) Scene Selection Flexibility (*n* = 2, 18.2%); (x) Improve the Comfort and Fit of Headset Devices (*n* = 6, 54.5%); (xi) Recommendation for Short VR Session to Prevent Fatigue and Overstimulation (*n* = 3, 27.3%); (xii) Integration of Hypermedia Features (*n* = 6, 54.5%); and (xiii) Accessibility Features (*n* = 4, 36.4%).

Recurring design recommendations across the studies pointed to the need for optimizing VR systems to meet the specific needs of PwCI. Rather than isolated technical suggestions, a coherent set of priorities emerged: improving visual and audio quality to enhance realism, simplifying navigation and controls, and offering alternative interaction methods such as voice commands or tangible interfaces. The inclusion of personalized reminiscence content and multisensory elements was also frequently highlighted as a means to deepen emotional impact. Importantly, the presence of trained facilitators was consistently linked to improved usability and user comfort. These insights form a foundation for refining future VRRT tools toward greater therapeutic accessibility and effectiveness.

#### 3.3.8. Theme 8: Limitations and Recommendations for Future Studies

Table A11 (Appendix B) outlines the limitations identified in current VR studies and provides recommendations for future research. A key limitation discussed is the challenge of achieving long-term cognitive and behavioral changes, as many studies report that the positive effects of VR interventions often do not persist beyond the duration of the sessions. This emphasizes the need for strategies to ensure sustained benefits over time. Another limitation is the presence of excessive motion in VR experiences, which can lead to discomfort or disorientation, suggesting the need for motion control adjustments to improve user comfort and accessibility. Results also highlights the importance of ensuring realism and engagement in VR design, as realistic and relatable environments are crucial for fostering emotional and cognitive connections. The lack of control groups in many studies is identified as a methodological weakness, making it difficult to attribute observed outcomes solely to VR interventions. This underscores the need for improved study designs that include appropriate comparisons to enhance the reliability of findings. Selection biases are another noted issue, with participant samples often being non-representative, which limits the generalizability of results. The table suggests inclusive recruitment strategies to address this. It also emphasizes the value of multi-session interventions, which can reinforce learning, improve retention, and yield more robust cognitive and behavioral outcomes compared to single-session studies. The reliance on self-reported measures is critiqued, as these can be subjective and prone to bias. Incorporating objective metrics or third-party evaluations is recommended to provide a more comprehensive understanding of VR’s effects. The table also discusses the need for improving user interaction mechanisms and simplifying navigation to enhance accessibility, particularly for individuals unfamiliar with VR or those with physical limitations. Further recommendations include increasing sample sizes to improve statistical power and reliability, addressing staffing challenges to facilitate VR session delivery, and ensuring baseline equality among participants to strengthen the validity of comparisons. The table also suggests exploring alternative interaction methods, such as voice commands, to reduce cognitive and physical demands, and developing systems that function effectively without constant internet access to enhance usability in under-resourced settings. These findings provide a roadmap for overcoming existing challenges and improving the effectiveness of VR interventions in future research. Here, the main extrapolated subthemes are summarized: (i) Lack of Long-Term Cognitive and Behavioral Change (*n* = 4, 36.4%); (ii) Addressing Excessive Motion in VR Experiences (*n* = 1, 9.1%); (iii) Ensuring Realism and Engagement in VR Design (*n* = 3, 27.3%); (iv) Lack of Control Group (*n* = 4, 36.4%); (v) Selection Biases (*n* = 4, 36.4%); (vi) Adding VR Sessions (multi-session interventions), (*n* = 10, 90.9%); (vii) Limitation of Self-Reported Measures (*n* = 4, 36.4%); (viii) Improving Interaction to Facilitate the Navigation of the VRs Themselves (*n* = 2, 18.2%); (ix) Improving Sample Size (*n* = 9, 81.8%); (x) Promoting Staff Availability to Facilitate Sessions and Time Constraints that Make it Difficult to Implement VR during Acute Behavioral Episodes (*n* = 3, 27.3%); (xi) Limited Baseline Equality (*n* = 3, 27.3%); (xii) Explore Alternative Interaction Methods (*n* = 3, 27.3%); and (xiii) Allow Accessibility without Internet (*n* = 1, 9.1%).

While the reviewed studies underscore the promise of VRRT, several methodological and design-related limitations were consistently noted. These included small sample sizes, lack of control groups, reliance on self-reported outcomes, and short intervention durations. Importantly, qualitative reflections across the studies emphasized the need for longitudinal designs, diverse recruitment strategies, and the integration of objective outcome measures. There was also a call for improving user interaction mechanisms and ensuring accessibility in low-resource settings. Collectively, these insights reinforce the necessity of refining both the research framework and technological infrastructure to advance VRRT as a reliable and inclusive therapeutic approach.

#### 3.3.9. Cross-Thematic Insights and Relationships

Beyond the individual findings described in each theme, the analysis highlights how the effectiveness and feasibility of VR-based reminiscence therapy (VRRT) emerge from the interplay of multiple, interdependent factors. For instance, participant engagement and emotional involvement (Themes 1 and 3) were closely linked to aspects such as system usability (Theme 2), the emotional resonance of the content (Theme 6), and the quality of immersion supported by the technical design (Theme 7). When users reported feeling present and emotionally connected during VR sessions, they were also more likely to demonstrate cognitive stimulation (Theme 4), particularly in cases where the content was personally meaningful or multisensory. At the same time, many of the challenges identified (Theme 5), such as discomfort with head-mounted displays or difficulties in navigation, were described as barriers that could reduce engagement and therapeutic impact if not properly addressed. These issues were often echoed in the limitations of existing studies (Theme 8), where methodological inconsistencies and short-term protocols made it difficult to draw firm conclusions about long-term benefits. A recurring insight across several themes was the importance of the facilitator—not only for technical support, but also as a relational presence that helped participants feel safe and supported during the sessions. This suggests that future implementations of VRRT should not only focus on improving hardware and software, but also consider how human support roles are integrated into the therapy experience. In summary, the themes identified in this review should not be interpreted in isolation. Rather, they reflect a complex and interconnected landscape in which user experience, emotional resonance, technical design, and therapeutic outcomes are all deeply intertwined. Recognizing and addressing these relationships is essential for advancing VRRT as a meaningful, person-centered intervention in cognitive and dementia care.

## 4. Discussion

### 4.1. Discussion Overview

The current scoping review intended to provide insights into the feasibility, usability, and effectiveness of VRRT for PwCI. The general findings suggest that VRRT enhances emotional well-being, engagement, and social interactions, e.g., [13,17,29]. While few studies have reported significant cognitive improvements, particularly in memory recall or preserving cognitive functioning, e.g., [8,17,29,31], others have found no measurable cognitive benefits, highlighting the variability in the effectiveness of VR, e.g., [30]. These findings suggest that while VR technology may enhance user engagement, its impact on cognitive outcomes remains inconsistent and more standardized methodologies are needed to accurately assess its efficacy. These discrepancies in cognitive results may be explained by several underlying factors. Notably, there was considerable heterogeneity across studies in terms of methodological design (e.g., randomized controlled trials vs. exploratory feasibility studies), participant characteristics (e.g., degree of cognitive impairment, prior VR experience), and intervention parameters such as duration, frequency, and content personalization. Furthermore, the cognitive domains assessed and the tools used to measure them varied widely, limiting comparability across studies. In several cases, brief or single-session interventions may not have been sufficient to generate measurable cognitive effects. Collectively, these factors likely contributed to the inconsistent findings observed, underscoring the need for more standardized and rigorously designed studies to evaluate VRRT’s cognitive impact.

The data indicate that VRRT has a positive emotional impact, particularly in alleviating anxiety and depression while fostering joy and relaxation, e.g., [8]. Key contributing factors include the recall of positive memories, strengthened emotional connections, and increased engagement in immersive environments, e.g., [17].

The thematic analysis and statistical findings highlight the importance of facilitators in VRRT implementation e.g., [16]. Given that facilitators play a key role in ensuring participant engagement and navigating technical challenges, future studies should focus on refining facilitator training protocols and assessing their impact on therapeutic outcomes [16]. Furthermore, the imbalance in the role assignment, where researchers (*n* = 7) commonly acted more as facilitators than healthcare professionals (*n* = 2), suggests the need to transition facilitation responsibilities toward caregivers to enhance real-world applicability. In line with thematic recommendations from studies addressing staff challenges in facilitating VR sessions, we acknowledge the necessity for actors beyond the patients themselves to be involved in the application’s usability mechanics. This is supported by (1) a significant number of sources discussed within this work (*n* = 3) that explicitly required proxies in their experimental procedures to perform meaningful interactions previously described; (2) thematic analysis indicating that less intuitive operations, such as navigating web applications, are preferably delegated to facilitators; and (3) extensive scientific literature on VR-based treatments for older adults and cognitively impaired individuals underscores the value of support personnel, who can assist in mitigating digital illiteracy among elderly users [34] and play a crucial role in managing potential adverse effects, such as dizziness or disorientation, while also adjusting VR settings as required [35,36]. We argue that future studies should pay greater attention to the role of the facilitator, their specific needs, and the potential risk of introducing experimental bias (referring to the methodological discussion lacking details on patient–facilitator interactions). Additionally, minimizing the researcher’s involvement is crucial to bridging the gap between experimental facilitation and the expected role of the final facilitator in real-world applications, e.g., [32].

Most of the reviewed studies demonstrated an awareness of participant accessibility needs, as evidenced by the requirement for seated or lying-down VR experiences in 54.5% of studies. Additionally, 72.7% of studies identified caregivers, family members, or researchers as “supporters”, reinforcing the necessity of assistance during VR sessions, e.g., [30]. However, while simplified controls and interfaces were recommended to improve usability, minor discomforts (such as eyestrain) were reported, e.g., [30]. Future research should explore alternative interaction methods, such as voice commands or gaze-based navigation, to enhance accessibility. Furthermore, thematic analysis identified key accessibility considerations: (1) simplified controls and interfaces were recommended to enhance accessibility, e.g., [15]; (2) reports of minor discomfort or eyestrain indicate a need for flexible session structures, e.g., [30]; and (3) accommodations for cognitive and physical challenges were identified as essential for improving participant experience, e.g., [13].

Another valuable finding is that even if the customization and personalization of contents have an independent impact on the disorder that participants have [37,38], approximately half of the selected studies (45.5%) integrated customized content based on the participants’ personal history, e.g., [8,15,16,18]. This is likely mainly due to the logistical challenges associated with acquiring and integrating personalized materials, e.g., [15,19,27,32]. However, even non-personalized reminiscence content was found to facilitate memory recall and conversation, suggesting that VRRT does not necessarily require individualized material to be effective. Instead, interactive elements that promote immersion and engagement may be key to optimizing reminiscence therapy outcomes. Qualitative findings suggest that RT experiences utilizing non-personalized materials still provide opportunities for dialogue and recollection. In fact, the remaining six studies qualitatively demonstrate the feasibility of the therapy, as patients tend to recall tangential memories related to their generic experience, e.g., [18]. From a scalability perspective, as VRRT solutions transition toward commercial applications, implementing therapy with generic VR content will likely be more cost-effective and market-ready than incorporating tools for uploading personalized audio or video, thereby facilitating widespread adoption.

While the therapeutic value of personalized content in VR reminiscence therapy (VRRT) has been highlighted by several studies [8,15,16,18,37,38], the implementation of such approaches remains technically and logistically demanding. Creating individualized VR experiences requires sourcing user-specific media (e.g., family photographs, culturally relevant audio), securing consent for use, and adapting content formats for immersive environments. This process is often resource-intensive, posing challenges in scalability, data management, and privacy. In clinical settings like nursing homes, where staff resources and time are constrained, this customization may not be feasible without dedicated technical support and streamlined content creation tools. In contrast, generic content—such as virtual recreations of historical landmarks or nature scenes—offers greater ease of deployment and lower production costs. These materials are often prepackaged, widely accessible, and adaptable to multiple users. Despite lacking individual specificity, generic VR content has been shown to trigger memory recall and emotional engagement through thematic relevance or cultural familiarity [18,30]. However, differences in efficacy between personalized and generic content merit deeper exploration. Personalized content may enhance emotional salience, increase cognitive stimulation, and foster a stronger sense of self-continuity, which can be particularly beneficial for individuals with moderate to severe cognitive impairment. On the other hand, generic content may suffice for early-stage cognitive decline or in group-based interventions where personalization is impractical. We recommend that future studies systematically compare the therapeutic outcomes of personalized versus generic VRRT across varied settings (e.g., institutional vs. home-based care). Mixed-methods research incorporating both quantitative outcome measures and qualitative user feedback will be critical in evaluating the cost–benefit ratio of personalization. Furthermore, the development of semi-automated personalization systems—potentially leveraging AI for user profiling and content matching—could offer a viable compromise between efficacy and scalability.

The level of interactivity of the system plays a crucial role in the effectiveness of VR reminiscence therapy, as it is directly proportional to overall engagement in the experience. Studies like [21] showed how the interactive dimension of VR experiences can moderate the effects of social presence, enhancing positive affect. Moreover, our thematic analysis indicates that increased interactivity exposes patients to a broader range of potential memory triggers, thereby enhancing therapeutic outcomes, e.g., [15]. The necessity for alternative interaction mechanisms is emphasized, particularly for individuals who encounter difficulties with conventional touch- or motion-based controls. Proposed approaches include more active engagement with virtual objects and scenarios to deepen immersion and enrich the overall experience, the use of tangible interfaces, voice-activated commands, and streamlined input devices to enhance accessibility for older adults and individuals with physical or cognitive limitations. A recurring recommendation also involves simplifying navigation systems to ensure that VR controls remain intuitive and user-friendly, thereby minimizing frustration and cognitive overload [15].

The variability observed across studies highlights the need for more standardized methodologies to accurately assess VRRT’s efficacy, e.g., [8,31]. The absence of control groups in many studies is recognized as a methodological limitation, complicating the attribution of observed effects solely to VR interventions. This highlights the necessity for more rigorous study designs incorporating appropriate comparison groups to strengthen the reliability of the findings. Selection bias is another concern, as participant samples are often non-representative, restricting the generalizability of results, which suggests that more inclusive recruitment strategies should be pursued. The benefits of multi-session interventions are also highlighted, since they can enhance learning, improve retention, and produce more robust cognitive and behavioral outcomes compared to single-session studies. Furthermore, the reliance on self-reported measures is criticized due to their subjective nature and susceptibility to bias. To achieve a more comprehensive assessment of VR’s effects, the integration of objective metrics or third-party evaluations is recommended. Future research should address this by adopting uniform evaluation criteria and well-defined control conditions.

### 4.2. Limitations

The selected studies showed significant limitations, mainly related to small sample sizes, lack of control groups, and limited long-term cognitive outcome data. These constraints hinder the ability to draw definitive conclusions regarding the sustained efficacy of virtual reality reminiscence therapy (VRRT). In more detail, the main barriers to VRRT identified were as follows:

- Technical Complexity: Older adults, particularly those with cognitive impairments, often face challenges in navigating VR environments due to the intricate nature of the technology. This underscores the necessity for user-friendly interfaces, simplified controls, and structured facilitator support to enhance accessibility and usability, e.g., [16,19].
- Physical Comfort: The prolonged use of head-mounted displays (HMDs) has been reported to cause discomfort, dizziness, or fatigue in some individuals. These issues highlight the need for advancements in hardware ergonomics, such as lighter and more adjustable headsets, as well as alternative display methods to improve the overall user experience, e.g., [30].
- Lack of Long-Term Data: Most studies focused primarily on short-term cognitive and emotional outcomes, making it difficult to determine whether VRRT offers enduring benefits. Future research should incorporate longitudinal designs to assess the sustained impact of VRRT on cognitive function, emotional well-being, and quality of life, e.g., [8,30].
- Study Design Limitations: Many investigations suffered from methodological weaknesses, including small and often non-representative sample sizes, the absence of control groups, and an overreliance on self-reported measures. These factors limit the generalizability and reliability of findings. To address these concerns, future studies should employ more rigorous experimental designs, include diverse participant populations, and incorporate objective assessments alongside self-reports to obtain a more comprehensive evaluation of VRRT’s effectiveness, e.g., [15,30].

Addressing these limitations will be crucial for refining VRRT applications and ensuring their efficacy as a therapeutic tool for PwCI.

### 4.3. Practical Implications and Recommendations

The findings of this scoping review have several important clinical and practical implications for the integration of VRRT into clinical settings for PwCI. Based on the summary of the primary results of the current review, the following evidence-based recommendations are proposed for healthcare professionals and implementation stakeholders:

- Facilitator Training and Role Transition: Develop standardized training programs for clinicians, therapists, and caregivers to conduct VRRT sessions. Transitioning facilitation duties from researchers to trained healthcare staff can increase ecological validity and support sustainable implementation in routine care settings.
- Integration into Routine Clinical Pathways: Incorporate VRRT into standard therapeutic schedules, particularly in geriatric or memory care programs. Structured sessions (e.g., 20–30 min weekly) can complement non-pharmacological cognitive stimulation therapies.
- Utilization of Thematic, Non-Personalized Content: Begin with curated, non-individualized VR experiences (e.g., cultural landmarks, historical events, nature scenes), which are easier to deploy and still effective in promoting reminiscence and emotional engagement.
- Technology Adaptation for Accessibility: Select ergonomic VR equipment and implement user-friendly controls such as gaze-based or voice-activated interfaces. Monitor participant responses to minimize discomfort and adjust session duration accordingly.
- Interdisciplinary Collaboration: Foster collaboration between clinicians, engineers, and researchers to co-develop tailored VRRT platforms that meet clinical and patient-specific needs.
- Addressing Psychosocial Dimensions: Leverage VRRT not only for cognitive stimulation, but also to enhance emotional well-being and reduce social isolation. Group-based VR interventions may facilitate peer interaction and increase social engagement in institutional settings.
- Stakeholder-Informed Feasibility Pilots: Before large-scale implementation, conduct pilot programs involving both patients and stakeholders to assess usability, staff burden, and logistical feasibility. These pilots can inform iterative improvements and guide implementation protocols.

These recommendations are intended to support the integration of VRRT into evidence-based clinical care, contributing to its development as an accessible, scalable, and therapeutically valuable tool for PwCI. However, it is important to emphasize that these implications must be interpreted with caution, as the current evidence base is still limited. Robust, large-scale studies—particularly randomized controlled trials with standardized protocols—are needed to further validate these recommendations and delineate more definitive clinical guidelines.

### 4.4. Future Research Directions

To advance the use of VRRT for PwCI, future research should address several critical areas, with a stronger emphasis on the development of personalized VR interventions and clearer pathways for clinical application:

- Larger, Controlled Studies: Future investigations should prioritize well-designed randomized controlled trials (RCTs) with larger and more diverse sample populations. Increasing the statistical power of these studies will help establish the efficacy of VRRT and ensure that findings are generalizable across different demographic groups. Additionally, incorporating multi-site trials could enhance the robustness and applicability of the results.
- Personalized Content Development: Several studies have noted the impact of individualized content on therapeutic engagement and emotional outcomes [8,15,16,18]. Personalized reminiscence materials, such as familiar photographs, culturally relevant locations, or personally meaningful themes, may significantly enhance memory recall and user satisfaction. However, logistical challenges persist in sourcing and integrating such content. Future research should investigate scalable personalization strategies, such as AI-assisted content customization or semi-automated user profiling, to balance therapeutic value with feasibility in clinical contexts.
- Alternative Interaction Methods: Investigating alternative interaction techniques is crucial for enhancing accessibility, particularly for individuals with motor or cognitive impairments. Future studies should assess the feasibility and effectiveness of gaze tracking, voice commands, gesture-based controls, and other adaptive interfaces to ensure that VRRT remains inclusive and user-friendly for all participants, regardless of their physical or cognitive limitations.
- Longitudinal Studies: Given the predominance of short-term studies in the current literature, there is a pressing need for long-term research examining the sustained effects of VRRT. Future studies should explore its impact on cognitive decline, emotional well-being, social engagement, and caregiver burden over extended periods. Longitudinal assessments will provide deeper insights into whether VRRT can contribute to slowing cognitive deterioration and improving overall quality of life for both patients and their caregivers.
- Feasibility Study with Patients, Caregivers, and Stakeholders: Given that our primary objective was to highlight qualitative data on the feasibility of reminiscence therapy studies in VR for PwCI, we also included experiments in which the sample consisted not of patients with cognitive impairment, but of stakeholders or caregivers involved with PwCI [15,16,19,32]. These participants tested the procedure and provided insights into the feasibility of using the proposed technological setups for individuals with cognitive decline. Future feasibility studies should deploy mixed-sample studies incorporating both individuals with cognitive impairment and stakeholders or caregivers to gather more comprehensive and informative data, ultimately supporting the design of future research focused on evaluating the effectiveness of these interventions, more deeply investigating the clinical practical implications, barriers, and opportunities to administer RT based on virtual environments.
- Exploring Immersive Multimodalities for Enhanced Reminiscence Therapy: Future research should implement studies to investigate the feasibility and effectiveness of immersive multimodalities, such as audio, video, haptic feedback, and scent, in enhancing RT. These heterogeneous stimuli could have the potential to create more engaging sensory experiences that may trigger memories more effectively and improve emotional well-being. Additionally, studies should explore the customization of these modalities based on individual preferences and cognitive conditions, as well as assess their long-term impact on mental health, quality of life, and therapeutic engagement. Moreover, effectiveness and practicality should be tested in both controlled and real-world settings.

By addressing these research gaps, future studies can contribute to the refinement of VRRT methodologies, ensuring that this emerging therapeutic approach becomes a more effective, accessible, and evidence-based intervention for PwCI. Importantly, efforts to personalize VRRT—despite their complexity—are crucial to maximizing therapeutic potential and aligning interventions with the lived experiences of individuals with cognitive impairment.

## 5. Conclusions

Virtual reality reminiscence therapy (VRRT) presents a promising approach for enhancing cognitive and emotional well-being in PwCI. However, current research is limited by small sample sizes, the absence of control groups, and a lack of long-term data, which restricts the generalizability of the findings. Key challenges include technical complexity, physical discomfort associated with head-mounted displays, and the need for personalized content to maximize therapeutic benefits. Future research should prioritize large-scale, randomized controlled trials, the development of personalized reminiscence content, and the exploration of alternative interaction methods to improve accessibility. Additionally, longitudinal studies are essential to assess the sustained impact of VRRT on cognitive decline, emotional well-being, and caregiver burden. Addressing these gaps will be crucial for refining VRRT applications and ensuring its efficacy as a viable therapeutic tool for individuals with cognitive impairments. 

## Figures and Tables

**Table 1:** Description of the eligibility criteria.

Eligibility Criteria Rationale	Eligibility Criteria Applied
The 1st Round eligibility criteria relied on **content-independent filters**, mostly pre-validated by the search strategies used, to ensure retrievability of records and consistency in filtering across databases with varying search filter granularity.	**1st Round:** **Inclusion criteria** **[CI_1.1]** English language. **[CI_1.2]** Only peer-reviewed articles (full peer-reviewed conference articles are included). **[CI_1.3]** No restrictions on publication date. **Exclusion criteria** **[CE_1.1]** Non-English. **[CE_1.2] Article types**: Book, book chapters, dissertations, abstracts, posters, interviews, protocols, reports. **[CE_1.3]** Non-original articles: meta-analysis and reviews. **[CE_1.4]** No full text available.
In the 2nd Round, the eligibility criteria were defined to narrow the selection of articles to those aligned with our conceptual framework while minimizing false negatives, given that researchers limited the screening at this stage to the **title and abstract only**. Consequently, the focus at this point was on mnemonic processes rather than reminiscence therapy, as prior research indicates that not all relevant studies explicitly mention reminiscence therapy, even when they employ its methodologies.	**2nd Round:** **Inclusion criteria** **[CI_2.1] Domain**: Digital mental health therapy focusing on cognitive impairment. **Exclusion criteria** **[CE_2.1]** No reference to therapeutic interventions for people with age-related cognitive impairments, mild or severe. **[CE_2.2]** No reference to any digital artifacts supporting interventions. **[CE_2.3]** No reference to treatments involving mnemonic processes.
In the Inclusion Round, these criteria were established to finalize the inclusion of articles in the study and to eliminate any false positives, relying on **full-text evaluation**. Our approach aims to encompass both primary targets and proxies and accept studies employing qualitative, quantitative, and mixed methods to comprehensively capture diverse aspects of reminiscence therapy and its integration into clinical practice.	**Inclusion round:** **Inclusion criteria** **[CI_3.1]** Only works that use either potential direct recipients of treatments or their proxies **†** as participants of an experiment. **[CI_3.2]** The work studies designing or utilizing one or more technologies implemented in VR meant to support interventions. **[CI_3.3]** The study concerns interventions in alignment with recognized frameworks such as reminiscence therapy or life recall. **Exclusion criteria** **[CE_3.1]** Studies without participants. **[CE_3.2]** Studies in which the direct recipients of treatments or, where proxies **†** are used, the individuals they represent do not have a diagnosed age-related cognitive impairment, mild or severe, assessed by an authority. **[CE_3.3]** The article does not report any qualitative or quantitative participant involvement results.

*Proxies* **†**: Within the previously outlined conceptual framework and guided by the criteria detailed in the accompanying table, we identified proxies as individuals designated as ***hetero evaluators*** for the purposes of this study. These proxies were deemed both competent and authorized to assess the effectiveness and impact of the therapy on the recipients.

**Table 2:** General information summaries of selected studies.

References	Study Design	Background	Objective(s)
Abdalrahim et al. [17]	Cross-sectional and longitudinal study	VRRT as a non-pharmacological approach to reduce apathy, enhance cognitive function, and improve mental well-being in dementia patients.	**Effectiveness** (of VRRT in reducing apathy, anxiety, and depression while enhancing cognitive function in older adults with dementia).
Afifi et al. [32]	Feasibility/longitudinal/mixed-methods study	Use of virtual reality (VR) to improve social engagement and relationships between PwCI and their family.	**Feasibility** (of using VR’s long-distance networking features with PwCI with a family member who lives at a distance, comparing their perceptions. Comparing the use of VRRT for people with mild cognitive impairment (MCI) and people with mild-to-moderate dementia).
Brimelow et al. [29]	Feasibility and mixed-methods study	VR as a non-pharma intervention for improving mental well-being, specifically reducing apathy and enhancing mood in aged care residents.	**Feasibility and preliminary data effectiveness** (of a fully immersive VRRT on apathy, mood, and engagement in aged care residents).
Coelho et al. [18]	Feasibility/longitudinal/mixed-methods study	Virtual Reality as a reminiscence therapy tool to improve engagement, evoke positive memories, and manage neuropsychiatric symptoms in dementia.	**Feasibility** (of promoting reminiscence utilizing VR headsets) **Effectiveness** (exploring the effects of VRRT for PwCI on measurable symptoms, degree quality of life supported by caregivers’ perspectives).
Huang et al. [8]	Longitudinal observational study	Novel non-pharma therapies for dementia, VRRT proved to improve anxiety, apathy, and cognitive function after usage with older adults. But there is lack of evidence for long term effects on people with dementia.	**Effectiveness** (of I-VRRT in older adults with dementia both immediately after and 3–6 months after intervention).
Saredakis et al. [13]	Feasibility and mixed-methods study	Non-pharmacological intervention using VR (Virtual Reality) for apathy management in aged care.	**Feasibility and preliminary data effectiveness** of VRRT (to reduce apathy and enhance cognitive engagement in aged care residents).
Saredakis et al. [30]	Non-randomized controlled trial	VR as a non-pharmacological tool for reminiscence therapy to reduce apathy and enhance emotional and cognitive engagement in older adults.	**Effectiveness** (comparing VRRT using HMDs with laptop-based and passive usual care for reducing apathy in residential aged care residents).
Siriaya et al. [19]	Feasibility/longitudinal/case design/mixed-methods study	Multi-faceted approach to dementia care, integrating person-centered social interaction methods with advanced technologies like virtual worlds and interventions of reminiscence therapy. This combination aims to enhance engagement, selfhood, and emotional well-being.	**Feasibility** (exploring how older people with dementia engage with virtual worlds; co-design a virtual worlds experience to encourage sustained lucid experiences with residents).
Sun et al. [15]	Exploratory/feasibility/mixed-methods study	Non-pharmacologic treatments (NPTs) for PwCI via immersive RT (FIRT) therapy.	**Feasibility** (of integrating VRRT into the Alzheimer’s Society’s existing dementia care program in the Durham Region of Ontario, Canada). **Enhance** the existing VRRT **prototype** (aiming for a comprehensive suite of assistive non-pharmacological tools to promote cognitive stimulation, reduce caregiver burden, and improve the quality of life for PwCI).
Sun et al. [16]	Exploratory/feasibility/mixed-methods study	Non-pharmacologic digital treatments for PwCI, web-based and non-immersive RT.	**Feasibility and effectiveness** (comparing caregiver of PwCI perceptions of web-based NI-VRRT vs. I-VRRT tools).
Tominari et al. [31]	Longitudinal/open-label/randomized controlled trial	Use of non-pharmacological interventions, particularly reminiscence therapy and virtual reality, to improve cognitive function and quality of life for people with dementia.	**Effectiveness** (comparing the VRRT-360 panoramas and RT utilizing digital not VR materials from the standpoints of cognitive functions, subjective well-being, and ADL in older adults with cognitive impairment).

Notes: To ensure terminological consistency, and given that samples of the included studies primarily consisted of individuals with mild cognitive impairment (MCI) and dementia, we have chosen to use the more general term “patients with cognitive impairment” where appropriate. In this study, this umbrella term specifically refers only to individuals diagnosed with MCI or dementia.

**Table 3:** Study outcomes.

**Study, Year**	**Variables Investigated**	**Experimental Results**	**Study Conclusions**
Abdalrahim et al. [17]	Apathy, cognitive functions, anxiety, and depression	Apathy: Significant reduction in PEARS scores from 17.20 (SD = 5.89) pre-intervention to 11.15 (SD = 4.55) post-intervention (t = −13.18, *p* < 0.001). Cognitive Function: SLUMS scores increased significantly from 15.11 (SD = 5.30) to 19.70 (SD = 4.91) post-intervention (t = 11.40, *p* < 0.001). Anxiety: HADS anxiety scores decreased from 13.66 (SD = 4.20) to 8.23 (SD = 4.71) (t = −11.18, *p* < 0.001). Depression: HADS depression scores reduced from 13.62 (SD = 5.31) to 9.33 (SD = 4.81) (t = −8.89, *p* < 0.001).	The study demonstrated that VRRT is an effective and culturally appropriate intervention for improving the quality of life in elderly individuals with dementia in care homes, showing benefits in reducing apathy, enhancing cognitive function, lowering anxiety and depression, and increasing enjoyment and engagement during sessions.
Afifi et al. [32]	User satisfaction and perceptions Sense of presence Conversational and behavioral engagement	**1. Evaluation of user satisfaction and perceptions (scale 1–10):** Residents reported high levels of satisfaction (M = 9.21), interest, fun, ease of use, safety, willingness to use again, and recommendation. Discomfort, eye irritation, nausea, anxiety, and tiredness were low. No significant differences across VR sessions. Family members’ detailed results are provided in the full manuscript. Comparison (residents vs. family members): Residents reported slightly higher satisfaction and ease of use, and less fatigue and nausea, though differences were not statistically significant. **2. Evaluation of user sense of presence (5-point scale):** Residents showed high telepresence (M = 4.32), social presence, and copresence. No significant differences across sessions. Family members reported slightly lower presence scores. Comparison: Residents experienced significantly higher telepresence, social presence, and copresence than family members (all *p* < 0.05). **3. Comparing conversational and behavioral engagement (VR vs. Baseline):** Significant increases in conversational engagement, vocalics, facial expressions, and both human-coded and automated kinesics during VR sessions compared to baseline (all *p* < 0.05). **4. Comparing MCI and dementia conditions:** No significant differences in satisfaction, ease of use, discomfort, or human-coded engagement between groups. Significant differences: Residents with dementia experienced higher telepresence, social presence, and copresence; residents with MCI showed greater automated physical (kinetic) engagement (*p* = 0.047).	VR was found to be safe and enjoyable, with high satisfaction and usability reported by both residents and family members. Residents with dementia experienced greater immersion but less physical engagement than those with MCI. Reminiscence-based VR sessions enhanced conversational and behavioral engagement compared to baseline phone interactions.
Brimelow et al. [29]	Apathy, emotional response, enjoyment, physical and emotional discomfort, and reminiscence	Apathy Reduction: Significant reduction in apathy was observed (PEARS mean pre-score = 15.54, SD = 6.11; post-score = 11.38, SD = 3.93; Z = −2.818, *p* = 0.005. Emotional Response: No significant increase in fear or anxiety was observed; a trend toward increased pleasure and alertness was noted. Enjoyment: All but one participant expressed a desire to experience VR again, citing enjoyment of scenes, particularly nature-based content like beaches and animals.	The study highlights VR as a promising tool to improve mood, engagement, and social interaction in aged care.
Coelho et al. [18]	Engagement levels, psychological symptoms, quality of life, and caregiver-reported impacts on well-being	Engagement: 73.5% of participants showed high interest in exploring the VR environment, and 57.7% communicated spontaneously about memories. Neuropsychiatric Symptoms and Quality of Life: No significant changes in the Neuropsychiatric Inventory (pre = 9.2, post = 9.7, *p* = 0.90) or EUROHIS-QOL-8 scores (pre = 28.6, post = 29.2, *p* = 0.66). Simulator Sickness: Mild symptoms (e.g., eyestrain, fullness of head) in a few participants, no severe side effects.	VRRT is a feasible and effective intervention for individuals with dementia, offering emotional, behavioral, and quality of life benefits. While further research with larger sample sizes is needed to validate these findings, the study highlights the potential of VR technology in enhancing dementia care.
Huang et al. [8]	Overall cognitive function (attention, concentration, orientation, short-term memory, long-term memory, language abilities, visual construction, list-generating fluency, abstraction, and judgment) Cognitive impairment (orientation, immediate recall, calculation or attention, delayed recall, naming, repetition, 3-stage command, reading, writing, and constructional praxis) Manifestation and severity of dementia (memory, orientation, judgment and problem solving, community affairs, home and hobbies, and personal care) Depressive symptoms Caregiver burden	No changes in cognitive function, global status, and caregiver burden immediately after the VR intervention. Significant reduction in depressive symptoms (CESD; *p* = 0.008). Moreover, compared with the cognitive function immediately after VR, it kept declining 3–6 months after. No significant differences in the MMSE, CASI and its subdomains, CDR-SB, and ZBI scores before and immediately after VR intervention, while the scores for CESD significantly decreased from 6.15 (SD 5.73) to 3.15 (SD 4.26; *p* = 0.008). CASI score significantly decreased 3–6 months after VR, compared to immediately after VR (52.14, SD 15.71 vs. 57.50, SD 12.40; *p* = 0.03.	Immersive VR reminiscence may improve mood and preserve cognitive function in elderly patients with dementia during the period of intervention.
Saredakis et al. [13]	Apathy, cognitive engagement (verbal fluency), presence, enjoyment, side-effects	Participation rate: 85% (17 out of 20 invited participants enrolled). Side effects: 35% (6/17 participants) reported mild, temporary side effects, including dizziness, eyestrain, and nausea. Enjoyment: All participants rated the VR experience positively, with many expressing interest in repeating the session. Semantic verbal fluency: Significant improvement post-VR (mean pre-VR = 9.73, SD = 4.10; mean post-VR = 12.20, SD = 4.54), t(14) = −3.27, *p* = 0.006. Phonemic fluency: No significant change post-VR (mean pre-VR = 10.13, SD = 3.78; mean post-VR = 9.73, SD = 3.81), t(14) = 0.55, *p* = 0.59. Association between apathy and verbal fluency: Positive correlation between initial apathy scores and semantic verbal fluency improvement: r = 0.719, 95% CI [0.327, 0.900], *p* = 0.003	VR was well tolerated with high engagement, significant cognitive benefits in semantic verbal fluency (particularly for those with higher baseline apathy), but with a need for careful monitoring of side effects. VR could be an effective and innovative tool to address symptoms of apathy in older adults, provided its use is carefully managed to mitigate potential side effects.
Saredakis et al. [30]	Apathy, cognition, depression, quality of life, loneliness, user preferences, enjoyment, engagement, simulator sickness symptoms, perceived changes in social involvement, cognitive awareness, and behavior from staff perspective	Apathy: No significant difference in apathy reduction between VR and laptop groups; however, the passive control group showed higher apathy levels than the intervention groups (*p* = 0.03). User Preferences: 73% of participants preferred VR over a flat screen for reminiscence viewing (*p* = 0.004). Session Enjoyment: Enjoyment was high across both VR and laptop groups, with slightly higher scores in VR. Side Effects: Minimal side effects reported; no significant differences in simulator sickness scores between sessions. Mild Symptoms: Two participants experienced short-term symptoms (e.g., headache, heavy-headed feeling). Secondary Outcomes: No significant improvements were observed for cognition, depression, quality of life, or loneliness.	VRRT appears to be a promising and feasible intervention in aged care settings, offering an engaging and enjoyable experience for residents. Although no significant differences in apathy were found between intervention and control groups, the act of reminiscing—regardless of format—seemed beneficial. Participants favored I-VR over flat-screen versions, and minimal side effects were reported, confirming its safety for older adults. Both I-VR and flat-screen approaches proved effective, with I-VR showing potential for future use due to its immersive appeal.
Siriaya et al. [19]	User engagement with the virtual worlds designed	Qualitative findings (observations, interviews and focus group): (1) Suspension of Disbelief (residents often perceived VWs as real, demonstrating strong engagement and a sense of presence). (2) Active Participation (residents engaged in activities reinforcing the feeling of performing real actions) (3) Success in designing Sustained Ludic Experiences (bright colors, particle effects, and stimulating feedback successfully sustained residents’ attention, old music energized residents and ambient sounds enhanced their connection to the virtual worlds (VWs). (4) Mood Restoration (engaging with VWs helped residents shift to a more positive emotional state). Three main aspects of reminiscence were observed in the study: (i) reminiscence from the sense of place, (ii) virtual-object-based reminiscence, (iii) reminiscence from the motion (gesture-based interactions). Joint activities (VWs fostered collaboration and strengthened relationships between caregivers and residents). Limitations to direct future works: (1) excessive physical motion; (2) personally irrelevant places, objects, and activities; (3) negative memories triggered; (4) perception of workload from facility staff; (5) touchscreen and gesture interactions less accessible for some. Suggestions: leveraging more on tangible/physical interface to empower the resident less confident with touch-based ones.	VW can provide interactive experiences to promote a continuing selfhood and provide stimulation and engagement to promote a feelgood factor. Negative memories could be mitigated by playful design. VW could also be a “place” that allows staff and residents to foster personal relationship and trust, which may improve care.
Sun et al. [15]	Usability and feasibility, navigation challenges, sense of presence, feedback on immersive experience, emotional impact, cognitive engagement, Caregiver burden, challenges, recommendations	(1) Usability and Feasibility (SUS): Overall usability as above average, with a median score of 69, though PwCI had a lower mean score (53.3) compared to caregivers (80). VRRT is feasible for integration into dementia care programs with adjustments to improve ease of use for PwCI. Minimal assistance was required to operate the VR headsets, though simplifications to navigation and controls would enhance accessibility. (2) Immersion and Engagement: High levels of immersion were reported, with median scores of: Spatial Presence: 4/5. Social Presence: 5/6. Environmental Presence: 4/5. PwCI described the VR experience as “realistic” and emotionally enjoyable, fostering happiness and calmness. Immersion promoted cognitive recall, such as reminiscing with personalized photos and audio. (3) Emotional and Cognitive Impact: VRRT effectively engaged PwCI in meaningful and stimulating activities, contributing to cognitive stimulation and positive emotional experiences. Caregivers perceived VRRT as a potential tool to reduce caregiving burdens by offering a structured and engaging activity for PwCI. (4) Challenges and Recommendations: Complexity of hand controls for PwCI. Eyeglasses compatibility issues for older participants. Simplify controls or incorporate non-controller-based activities. Adjust headsets for better comfort and usability. Include more interactive and purposeful activities to sustain engagement.	Virtual reality reminiscence therapy is a promising innovation in dementia care, offering immersive, engaging, and potentially therapeutic experiences for PwCI while supporting caregivers. Further refinements are needed to enhance usability and maximize its impact.
Sun et al. [16]	Usability and efficacy, engagement and emotional impact, barriers and challenges, recommendations	(1) User-friendly and inclusiveness, client-centered and individualized approach to therapy, digitalization of reminiscence therapy, integrated and secured system, increased care participation, continuity of care. (2) Relationship building with PwCI, increases understanding of PwCI through personalized content, promotes better communication between caregivers and PwCI, reduces HCP’s workload, facilitates accessibility of reminiscence therapy. (3) Improves memory recall, quality of life, and mental well-being, alleviates negative behaviors, augments positive self-expression, reduces the risks of social isolation and wandering. (4) Improves social connection, family involvement in care, accessible in any setting. HCPs have indicated the significance of web-based RT in reducing behavioral and psychological symptoms of dementia. (5) Improves memory recall and mental well-being. (6) Alleviates negative behaviors. (7) Augments positive self-expression. (8) Reduces the risks of social isolation and wandering. (9) Improves social connection. (10) Promotes accessibility and family involvement in care. (11) Accessibility in any setting. Encourage involvement from younger individuals, offer training on the web app, and enhance the platform with features such as narrative descriptions for reminiscence, integration with other digital tools, QR code support, and a hybrid of digital and traditional approaches. **For web-based reminiscence therapy (RT):** Prevent image distortion during uploads; add a mobile upload option to leverage family members’ smartphones; allow bulk media uploads to save time; provide user support for system navigation; ensure accessibility across devices, not just desktops/laptops; offer tutorial videos to support user onboarding. **For non-immersive VR reminiscence therapy (NI-VRRT):** Improve visual realism to avoid the appearance of low-quality content; enable zoom for multimedia elements; simplify the visual environment by reducing on-screen objects; enhance audio playback quality; consider larger displays to improve immersion; provide training on using hotkeys; include clear, guided instructions to support ease of use. Average SUS score of 80/100 for the WebApp is 78.3/100 for the NIRT environment (considered usable with score above 68).	The data indicate that both web-based RT and NI-VRRT are viable tools for reminiscence therapy, with high usability scores and positive caregiver feedback. The recommendations for improving functionality and user-friendliness suggest opportunities to enhance their effectiveness in dementia care.
Tominari et al. [31]	Cognitive functions, subjective well-being, behavioral and cognitive observations, executive function, language and verbal fluency	1. Cognitive Function (MMSE Scores) VR Panoramas Group: (Baseline: 22.1 ± 2.4; Post-Intervention: 25.3 ± 2.1 (*p* < 0.001); Improvement: +3.2 points on average); Conventional Still Photos Group: (Baseline: 21.9 ± 2.6; Post-Intervention: 23.1 ± 2.3 (*p* < 0.05); Improvement: +1.2 points on average). 2. Subjective Well-Being (PGC Morale Scale) VR Panoramas Group: (Baseline: 13.6 ± 3.1; Post-Intervention: 17.8 ± 3.0 (*p* < 0.01); Improvement: +4.2 points); Conventional Still Photos Group: (Baseline: 13.4 ± 2.8; Post-Intervention: 15.2 ± 3.1 (*p* < 0.05); Improvement: +1.8 points). 3. Behavioral and Emotional Observations (MOSES Scores) VR Panoramas Group: Showed significant improvement in activity level, mood, and engagement. Participants were rated as “highly engaged” in 85% of sessions compared to 60% in the still photos group; Conventional Still Photos Group: Moderate improvement in mood and engagement, but less consistent than the VR group. 4. Executive Function (Trail Making Tests) TMT A (Processing Speed and Attention): VR Panoramas Group: (Baseline: 45.2 ± 9.8 s; Post-Intervention: 37.5 ± 8.6 s (*p* < 0.05); Improvement: −7.7 s); Conventional Still Photos Group: (Baseline: 46.1 ± 10.2 s; Post-Intervention: 42.4 ± 9.9 s (*p* > 0.05); Improvement: −3.7 s). Trail Making Test B (Cognitive Flexibility): VR Panoramas Group: (Baseline: 95.3 ± 15.1 s; Post-Intervention: 80.7 ± 14.4 s (*p* < 0.01); Improvement: −14.6 s; Conventional Still Photos Group: (Baseline: 97.2 ± 16.5 s; Post-Intervention: 92.8 ± 15.7 s (*p* > 0.05); Improvement: −4.4 s. 5. Language and Verbal Fluency (Word Fluency Test) VR Panoramas Group: Average number of words generated increased from 11.4 ± 2.3 to 14.7 ± 2.5 (*p* < 0.01); Conventional Still Photos Group: 9Average number of words generated increased from 11.2 ± 2.4 to 12.3 ± 2.6 (*p* > 0.05). 6. Participant Feedback (Participants in the VR Panoramas Group reported that the 360° images felt “immersive” and “brought back vivid memories”; The Conventional Still Photos Group described the experience as “pleasant but not engaging.”).	The results demonstrate that VR reminiscence therapy using 360° panoramas is more effective than conventional still photo therapy in improving cognitive function, subjective well-being, and engagement among older adults with mild dementia. The immersive nature of VR likely contributed to its superior outcomes. - The interactive and immersive nature of VR enhances cognitive stimulation, particularly in areas of memory recall, attention, and problem-solving. - The immersive VR environment appears to evoke stronger emotional connections and positive reminiscence experiences, contributing to better mood and overall psychological well-being. - VR therapy promoted more active participation and evoked richer discussions and memories. - Caregivers observed reduced agitation and increased happiness in participants after VR sessions. - VR appears to outperform traditional reminiscence approaches by leveraging its immersive qualities to evoke stronger emotional and cognitive responses.

Notes: To ensure terminological consistency and given that samples of the included studies primarily consisted of individuals with mild cognitive impairment (MCI) and dementia, we have chosen to use the more general term “patients with cognitive impairment” where appropriate. In this study, this umbrella term specifically refers only to individuals diagnosed with MCI or dementia.

## Data Availability

Data available in a publicly repository: https://doi.org/10.5281/zenodo.15182014, accessed on 8 May 2025.

## Supplementary Materials

The following supporting information can be downloaded at: https://doi.org/10.5281/zenodo.15182014, accessed on 8 May 2025.

## Abbreviations

The following abbreviations are used in this manuscript:

VR	Virtual Reality
PwCI	People with Cognitive Impairment
VRRT	Virtual Reality Reminiscence Therapy
PRISMA-ScR	Preferred Reporting Items for Systematic Reviews and Meta-Analyses Extension for Scoping Reviews
OSF	Center for Open Science
QoL	Quality of Life
RT	Reminiscence Therapy
NI-VRRT	Non-Immersive Virtual Reality Reminiscence Therapy
I-VRRT	Immersive Virtual Reality Reminiscence Therapy
HMD	Head-Mounted Display
PAS	Psychogeriatric Assessment Scale
AES	Apathy Evaluation Scale
SSQ	Simulator Sickness Questionnaire
SUS	System Usability Scale
ZBI	Zarit Burden Interview
CESD	Center for Epidemiological Studies Depression
CDR	Clinical Dementia Rating
CASI	Cognitive Abilities Screening Instrument
MMSE	Mini-Mental State Examination
MoCA	Montreal Cognitive Assessment
SLUMS	Saint Louis University Mental Status
HADS	Hospital Anxiety and Depression Scale
NPI	Neuropsychiatric Inventory
EUROHIS-QOL-8	European Health Interview Survey Quality of Life 8-item index
PGC	Philadelphia Geriatric Center
MOSES	Multidimensional Observation Scale for Elderly Subjects
TMT	Trail Making Test
WFT	Word Fluency Test
BPSD	Behavioral and Psychological Symptoms of Dementia
HCP	Health Care Provider

## Appendix A

**Table A1:** Demographic characteristics of participants in the experiments.

Study, Year	Single-Center /Multi-Center	Out/Inpatients	Inclusion/Exclusion Criteria	Recruitment Methods	Sample Size	Gender	Years of Age Mean (SD), [Range]	Cognitive or Dementia Conditions ^a^	Comorbid Diagnosis
Abdalrahim et al. [17]	Multi-center (*n* = 3)	IN	Inclusion criteria: - Patients with clinical dementia diagnosis who could consent (or had a consultee if they could not consent themselves). Exclusion criteria: - Those deemed unfit by care staff or those diagnosed with other psychiatric disorders.	Convenience sampling with eligibility assessed by care staff; consent obtained from participants or consultees.	*n* = 75 (PwCI)	*n* = 41 (female), *n* = 34 (male)	PwCI: 65.5 (2.2), [N/A]	Major neurocognitive disorder: dementia (unspecified types) (*n* = 75)	N/A
Afifi et al. [32]	Single-center	OUT	Inclusion criteria: - Residents of senior living community (Maravilla, Santa Barbara, CA) in August 2019 to March 2020 - Residents with MCI or mild-to-moderate dementia - Residents with family members living at a distance. Exclusion criteria: - Individuals with hallucinations, paranoia, aggression, vertigo, or abusive relationships.	Recruitment through letters, staff announcements, townhall meetings, and one-on-one discussions. Consent was obtained directly from participants and their legal representatives.	*n* = 21 (PwCI) *n* = 21 (Family members)	PwCI: *n* = 18 (female), *n* = 3 (male) Family members: *n* = 9 (female), *n* = 12 (male)	PwCI: 83.10 (9.76), [54–94] Family members: 59.86 (14.12), [18–83]	Mild neurocognitive disorder: *n* = 9 Major neurocognitive disorder: Mild: *n* = 4, Moderate: *n* = 8	N/A
Brimelow et al. [29]	Single-center	IN	Inclusion criteria: - Psychogeriatric Assessment Scale (PAS) score ≤4, indicating no or minimal cognitive impairment, with consent or guardian consent as appropriate. Exclusion criteria: - acute illness, contagious conditions, or inability to sit up.	Residents were informed of the VR program through advertising, and participation was opt-in with consent obtained.	*n* = 13 (PwCI)	*n* = 9 (female), *n* = 4 (male)	PwCI: 82 (8), [66–93]	Mild neurocognitive impairment: mild (*n* = 4) Major neurocognitive disorder: dementia (*n* = 9) (2 = moderate; 7 = severe)	N/A
Coelho et al. [18]	Multi-center (*n* = 2)	IN	Inclusion criteria: - adults aged 65 - having a dementia diagnosis - being able to recall memories with the aid of caregivers. Exclusion criteria: - severe visual impairments, advanced dementia stages, and diagnosis of specific psychiatric disorders (e.g., schizophrenia).	Convenience sampling in two local institutions, informed consent obtained from participants or guardians.	*n* = 9 (PwCI)	*n* = 6 (female), *n* = 3 (male)	85.6 (7.4), [N/A]	Major neurocognitive disorder: dementia (unspecified types) (*n* = 9). Moderate: *n* = 3 Moderately severe: *n* = 3 Severe: *n* = 3	N/A
Huang et al. [8]	Multi-center (*n* = 2)	IN	Inclusion criteria: - diagnosed with all-cause dementia by experienced physicians, based on the National Institute on Aging and Alzheimer’s Association diagnostic criteria - attended the dementia care unit between July 2020 and March 2021 Exclusion criteria: - they had poor recognition of the VR images even after adjusting the mounting position of the headset - their cognitive function was too low or their BPSD was too severe making assessment difficult.	Recruited from 2 dementia care units in Kaohsiung city, Taiwan, identified by the staff at the dementia care units based on the inclusion and exclusion criteria	*n* = 20 (PwCI)	*n* = 11 (female), *n*= 9 (male)	79.0 (7.8), [N/A]	Major neurocognitive disorder: dementia (unspecified types): very mild (*n* = 2), mild (*n* = 15), moderate (*n* = 3)	N/A
Saredakis et al. [13]	Single-center	IN	Inclusion criteria: - residents without severe cognitive impairment (assessed by Psychogeriatric Assessment Scale score <16) and those without agitation or vision impairments uncorrectable with glasses. Exclusion criteria: - presence of neurological disorders, severe cognitive decline, or physical limitations incompatible with VR use.	Participants identified by senior staff based on criteria; approached directly by researchers for consent.	*n* = 17 (PwCI)	*n* = 10 (female), *n* = 7 (male)	PwCI: 87.3 (6.3), [72–95]	Mild neurocognitive disorder (MCI): no or minimal (*n* = 10), mild (*n* = 3), moderate (*n* = 4)	Apathy (*n* = 3)
Saredakis et al. [30]	Multi-center (*n* = 3)	IN	Inclusion criteria: - residents aged 65+ years with moderate impairment (Psychogeriatric Assessment Scale score ≤ 15) - vision corrected to fit an HMD. Exclusion criteria: - for severe cognitive impairment, severe physical or mental conditions preventing VR use.	Participants recruited by senior facility staff based on eligibility; informed consent obtained by research staff.	*n* = 43 (PwCI)	*n* = 28 (female), *n* = 15 (male)	PwCI: 84.8 (8), [71–103]	Major neurocognitive disorder: up to moderate (PAS ≤ 15) (*n* = 43); in details: Memory-related, dementia, or Parkinson disease (*n* = 11)	Apathy (*n* = 28), Depression and anxiety (*n* = 16)
Siriaya et al. [19]	Multi-center (*n* = 2)	IN	Inclusion criteria: - residents of the 2 care homes involved in the study	N/A	*n* = 6 (Caregivers) *n* = 2 (Managers)	N/A	Caregivers/Managers: N/A (N/A), [80–101]	(referred to) Major neurocognitive disorder: dementia (unspecified types)	N/A
Sun et al. [15]	Single-center	IN	Eligibility screening - Assess cognitive functioning of PwCI with Mini-Mental State Exam cognitive assessment scores above 24 points for the Mini-Mental State Exam [4] and above 10 points for the Montreal Cognitive Assessment.	Purposive sampling was used to recruit individuals from the study setting (Participants’ interests were solicited through recruitment posters posted in the study setting)	*n* = 3 (PwCI) *n* = 7 (Health staff)	PwCI: N/A Staff: *n* = 5 (female), *n* = 2 (male)	PwCI: N/A (N/A), [65–74] Health staff: N/A (N/A), [25–84]	Major neurocognitive disorder: dementia (unspecified types) (*n* = 3)	N/A
Sun et al. [16]	Single-center	IN	Inclusion criteria: - HCPs (health care providers) who directly worked with PwCI - have experience managing BPSD (Behavioral and Psychological Symptoms of Dementia) - over the age of 18 years - capable of providing informed written consent and can speak and understand English	Ontario Shores Staff members realized and delivered a recruitment flyer. Patients were selected form 2 units: Geriatric Dementia Unit (GDU) and Geriatric Transitional Unit (GTU) of the Center of experimentation	*n* = 10 (healthcare providers)	Healthcare providers: *n* = 8 (female), *n* = 2 (male)	Healthcare providers: N/A (N/A), [18,59]	N/A	N/A
Tominari et al. [31]	Multi-center (*n* = 7)	IN	Inclusion criteria: - Japanese-speaking, aged 65 years or older - clinically diagnosed with dementia on the mini-mental state examination (MMSE) scores ranging from 22 to 26 - no other psychological disorders - no visual nor auditory impairments that would interfere with reminiscence therapy - no exposure to reminiscence therapy over the past three months - able to answer “Yes” or “No” to close-ended questions - able to view images for approximately 30 min - no demonstrated previous experience with tablet-type devices - selected the participants according to their attention span, which was judged by the nursery staff during the recreation and rehabilitation time	Participants were recruited from four elder-care facilities and three nursing homes in Japan PwCI expressed the intention to participate in this study.	*n* = 52 (PwCI)	*n* = 40 (female), *n* = 12 (male)	86.05 (N/A), [68–98]	Mild neurocognitive disorder	N/A

Notes: ^a^ https://pmc.ncbi.nlm.nih.gov/articles/PMC4104432/, accessed on 8 May 2025. Notes: To ensure terminological consistency, and given that samples of the included studies primarily consisted of individuals with mild cognitive impairment (MCI) and dementia, we have chosen to use the more general term “patients with cognitive impairment” where appropriate. In this study, this umbrella term specifically refers only to individuals diagnosed with MCI or dementia; N/A = Not Applicable.

**Table A2:** Characteristics of experimental methodology.

Study, Year	Source of Data	Other Experiment Actors	Interaction with the System During the Experiment	Conversating with Facilitators About RT Materials	Experimental Procedure	Measures	Data Collection Types
Abdalrahim et al. [17]	Auto-referred	Facility staff (recruiters), researchers (recruiters, assessors)	N/A	N/A	(1) Pre-Assessment phase: baseline data on apathy, cognitive function, anxiety, and depression. (2) VRRT sessions phase: 10 guided sessions, each lasting up to an hour, over five weeks (twice weekly). Sessions were one-on-one, conducted in Arabic, and designed to evoke individual or group “positive memories.” (3) Post-Assessment phase: Assessment of all outcomes immediately after the final VR session.	Saint Louis University Mental Status (SLUMS)—Arabic Version: Person-Environment Apathy Rating Scale (PEARS)—Arabic Translation: Hospital Anxiety and Depression Scale (HADS)—Arabic Version	Quantitative data collection: self-reported data, demographic and health data, behavioral data. Qualitative data collection: users and staff interviews.
Afifi et al. [32]	Mixed	Staff (recruiters), researchers (recruiters, facilitators, assessors), family members (support, RT material providers),	Participant and proxy interaction Residents and family members: observing their photos and video in a virtual family room, from seated position. Family member: input the addresses into the Rendever online portal Residents: chose avatars to represent themselves and their family member Researchers: operated the VR using a control tablet.	N/A	Baseline 15 min phone conversation. Weekly VR sessions over three weeks, average length 30.36 min. Featuring: (1) 1 of 25 Preprogrammed Virtual travel adventures. (2) Visits to favorite past addresses or destinations. (3) Viewing family photos/videos in a virtual family room. Residents experienced the VR in a private room at the senior living community while family members participated from their own homes. Data collection included surveys, behavioral observations, and automated analysis of kinetic engagement. Analysis: computed repeated measures analysis of variance (ANOVA), paired t tests, between subjects ANOVAs all test were two-tailed, with α = 0.05	Mini-Mental State Examination-2 (MMSE-2) Adapted questionnaire items about user satisfaction and perceptions (scale items) Adapted questionnaire items about sense of presence (scale items) Observational coding items from Relational Communication Scale about conversational engagement (scale items) Observational coding items about behavioral engagement (scale items) Post-Session Interviews	Quantitative data collection: self-reported data, demographic and health data, behavioural data, standardized cognitive screening tools, Qualitative data collection: Pre study interview, Debriefing interview
Brimelow et al. [29]	Mixed	Leisure staff, lifestyle staff (support), lifestyle Coordinator, personal carer and registered nurse (*n* = 2) (facilitators, assessors), researchers (assessors).	No or minimal interaction Participants—observation; posture N/A Leisure and lifestyle coordinator—set up the scenery for the experiment	Yes + reported example questions	(1) Pre-VR Observation: users were observed for 10 min to record baseline levels of apathy and emotion. (2) Setup: Facilitators (lifestyle coordinators or research assistants):—selected VR scenes based on resident preferences; -secured the headset on the resident and adjusted it for comfort; -facilitators explained the process and set expectations before starting the VR session. (3) VR Session: facilitators encouraged engagement by asking open-ended questions related to the scene: *“What do you see?”; “Have you been to a similar place?”*. Facilitators also monitored for symptoms of simulator sickness and provided instructions to mitigate discomfort, such as closing eyes before removing the headset. (4) Post-VR Observation: Users were observed for 10 min to measure apathy and emotions. Users were also asked about their experiences and preferences in a structured interview.	Person–Environment Apathy Rating Scale (PEARS) Observed Emotion Rating Scale (OERS) Structured Interviews for Qualitative Feedback. Simulator Sickness Questionnaire (SSQ) Emotion and Mood Assessment in Dementia Studies	Quantitative data collection: self-reported data, behavioral data. Qualitative data collection: resident feedback survey, staff interviews, observational notes.
Coelho et al. [18]	Mixed	Caregiver including family members (support), family members (RT material providers), researchers (facilitators, RT material providers, assessors)	No or minimal interaction Participants—observation from seated position	Yes + reported example questions	(1) Preparation of Intervention Personalization: Semi-structured interviews with participants and caregivers identified meaningful life memories, activities, and locations. Researchers used these details to create 360° video recordings of specific places, such as childhood homes, workplaces, or religious venues. Video Production: Filming conducted using a GoPro Fusion 360 camera, edited with Adobe Premiere Pro. High-resolution recordings ensured clear visualization, avoiding stressful stimuli (e.g., loud noises or obscured views). (2) Reminiscence Program Implementation Session Setup (Four individual sessions over two weeks. Conducted in either institutional facilities or a dedicated VR lab). VR Session: Participants viewed personalized 360° videos for a maximum of 15 min while seated in a rotating chair to explore the environment. Researchers guided participants using verbal cues, e.g., “What do you see?” or “Does this place make you remember anything?” (3) Post-Session: Participants discussed their feelings and shared recollections of the video. Simulator sickness symptoms were reassessed. Data Collection: Engagement and Behavior: Observations on interest in the 360° environment, communication, memory recall, and overall experience. Levels of interest rated as very interested, moderately interested, or not interested. Psychological and Behavioral Symptoms: Symptoms like anxiety, agitation, or irritability were recorded using a customized scale based on the Cornell Scale for Depression in Dementia and the Neuropsychiatric Inventory. Simulator Sickness: Symptoms like eyestrain, dizziness, or nausea were evaluated pre- and post-session using the Simulator Sickness Questionnaire. Pre- and Post-Intervention Assessments: Neuropsychiatric Inventory: Assessed dementia-related symptoms (score range: 0–144). EUROHIS-QOL-8: Measured perceived quality of life (score range: 8–40).	Neuropsychiatric Inventory (NPI) EUROHIS-QOL-8 Simulator Sickness Questionnaire (SSQ) Observation of Engagement Semi-Structured Interviews with Caregivers.	Quantitative data collection: Standardized cognitive screening tools, self-reported data, demographic and health data. Qualitative data collection: Focus groups
Huang et al. [8]	Auto-referred	Facility staff (recruiters), family and caregivers (RT material providers), researchers (support, facilitators)	Participant interaction Participants—controllers of interactions (play/pause, feed chickens) from seated position Researchers—observing mirrored viewport	Yes	(1) Before-Intervention Assessment: Cognitive function, Global status, Depressive symptoms, Caregiver burden. (2) VR Intervention (twice per week for 3 month, each session 10–12 min): Participants were seated to avoid motion sickness and fall risks. VR content was mirrored on a laptop for the researcher to monitor the participant’s interactions. Participants used controllers for interaction, with guidance and support from researchers when needed. (3) After-Intervention Assessment: Cognitive function, Global status, Depressive symptoms, Caregiver burden. (4) Long-Term Assessments: 3–6 months after intervention using the same measures.	Cognitive Abilities Screening Instrument (CASI) Mini-Mental State Examination (MMSE) Clinical Dementia Rating (CDR) Center for Epidemiological Studies Depression (CESD) Zarit Caregiver Burden Interview (ZBI)	Quantitative data collection: self-reported data, demographic data. Qualitative data collection: observational notes.
Saredakis et al. [13]	Auto-referred	Facility staff (support, recruiters), researchers (facilitators, researchers, assessors)	Proxy interacting Participants—observation from seated position Researchers—6DOF navigation from mirrored viewport	Yes	(1) Session 1: Demographic data collection and pre-study interviews to customize VR content. Participants viewed a brief VR demo to confirm suitability for the headset. (2) Content preparation: Tailored of the VR content (90 min per users) (3) Session 2: a 20 min VR reminiscence session using HMDs (-Pre-VR assessments: SSQ, verbal fluency tasks, and expectation ratings; -Participants viewed VR content for 20 min while discussing related memories with the researcher; -Post-VR assessments: SSQ, verbal fluency tasks, enjoyment ratings, and debrief interviews).	Apathy Evaluation Scale (AES) Psychogeriatric Assessment Scale (PAS) Simulator Sickness Questionnaire (SSQ) Presence Measures—Slater–Usoh–Steed Presence Questionnaire (SUS) Verbal Fluency Tests Qualitative Feedback—Debriefing Interviews	Quantitative data collection: self-reported data, performance-based tasks, demographic and health data. Qualitative data collection: Pre-study interview, debriefing interview.
Saredakis et al. [30]	Mixed	Senior facility staff (recruiters), researchers (RT material providers, support, recruiters, facilitators, assessors)	Proxy interacting Participants—observation from seated or lying position Researchers—6DOF navigation from mirrored viewport	Yes	Three groups: (1) VR-based reminiscence using Oculus Quest HMDs, (2) laptop-based reminiscence, and (3) usual care (control). Each intervention group underwent three 20 min reminiscence sessions over two weeks, with themed content (e.g., personal memories, nature scenes). Phases: (1) Baseline Assessment (60 min). Participants in the VR and active control groups: 60 min reminiscence interview to personalize intervention content. (2) Intervention (three individual reminiscence sessions within two weeks, each lasting 20 min). (3) pre- and post-session measures for VR participants. (4) Follow-Up Measures Conducted the day after the final intervention session. Repeated all baseline measures (AES, ACE-III, GDS, etc.). Qualitative feedback on the reminiscence experience was gathered.	Psychogeriatric Assessment Scales (PAS) Apathy Evaluation Scale (AES) Addenbrooke’s Cognitive Examination III (ACE-III) Geriatric Depression Scale (GDS) Quality of Life in Alzheimer’s Disease (QOL-AD) Three-Item Loneliness Scale Simulator Sickness Questionnaire (SSQ) Session Records: Assessed attendance, memory recall, responsiveness, interaction, and enjoyment during reminiscence sessions. Staff Questionnaire: Evaluated perceived changes in social involvement, cognitive awareness, and behavior.	Quantitative data collection: self-reported data. Qualitative data collection: resident feedback survey, staff interviews.
Siriaya et al. [19]	External observer data	Caregivers (facilitators, assessors), researchers (assessors, support)	Participant interacting Participants: interactions with upper limb movements (picking up items), from seated mode, could navigate with 6DOF	N/A	9 visits were made to 2 care homes, and 8 observation sessions were carried out over the period of eight months. After each visit where observation sessions were conducted interviews were carried out with the care staff. 2 focus group sessions were carried out with the care staff and managers. Analysis: thematic analysis	No quantitative measures employed Observation of Engagement Pre and Post Session Interviews with staff facility Focus Group discussions	Qualitative data collection: pre and post study not structured interviews, focus groups discussions, observational notes.
Sun et al. [15]	Mixed	Researchers (recruiters, facilitators, assessors)	Participant interaction Participants—selection play/pause of RT materials in VR, free navigating VR scenes, 3D painting activity; posture N/A	N/A	VR sessions: Participants used Meta Quest 3 VR headsets to experience VR environments. Tailored content included images, audio, videos, and interactive activities such as painting and virtual travel (10–15 min per session). Assessment timeline: (1) Pre-Intervention assessment: Conducted before participants were introduced to the VR demonstration. (2) During Intervention assessment: observations during VR demonstrations to assess usability and participant engagement. Initial usability of the VRRT prototype was tested during interactive sessions. (3) Post-intervention assessment: conducted immediately after the VR demonstration and usability testing.	Mini-Mental State Examination (MMSE) Montreal Cognitive Assessment (MoCA) Participant Demographic Information Short Form Zarit Burden Interview (ZBI-12) System Usability Scale (SUS) Presence Questionnaires Focus Group and Semi-Structured Interview	Quantitative data collection: Standardized cognitive screening tools, self-reported data, demographic and health data. Qualitative data collection: Focus groups, semi-structured interviews, observational notes.
Sun et al. [16]	External observer data	Facility staff (recruiters), care giver (RT material providers), family member (support), researchers (recruiters, facilitation, assessors)	Participant interaction Participants—navigate the VR environment, select and interact with RT materials (play/pause/volume); posture N/A Caregiver—indicate which media to display during execution	N/A	1) Setup: Caregivers and HCPs were introduced to both web-based RT and NI-VRRT systems. Content for sessions was selected and uploaded based on individual preferences or generic themes. 2) web-based RT and NI-VRRT Sessions: Conducted in dementia care units or caregiver facilities. Each session included the display of selected media and caregiver-patient interaction. 3) Data Collection: Participants completed the System Usability Scale (SUS) after each session. Feedback interviews were conducted to gather qualitative insights into usability and effectiveness.	System Usability Scale (SUS) to the caregivers. Interviews.	Quantitative data collection: self-reported data, demographic data. Qualitative data collection: semi-structured interviews, observational notes.
Tominari et al. [31]	Auto-referred	Trained nurse (recruiters, facilitators), researchers (RT material providers, assessors)	Proxy interaction Participants: observing; posture N/A Trained nurse: enlarged or reduced 360 image on tablet	Yes	(1) Intervention (8 weekly sessions per participant. 30–45 min per session) VR Panoramas Group: Used 9.7-inch iPads to display 360° VR panoramic images. Participants could enlarge, reduce, and adjust the viewing angles freely. Scenes included traditional Japanese settings, such as homes, schools, and cultural artifacts from the 1940s–1960s. Sessions were facilitated by a certified nurse trained in reminiscence therapy. Conventional Still Photos Group: Viewed printed panoramic photos of the same settings used in the VR group. Each panoramic scene was divided into four printed images covering 90° each. Facilitators prompted participants to reminisce and discuss the images they viewed during the sessions. (2) Assessments Baseline and Post-Intervention: Cognitive and behavioral assessment, subjective well-being.	Mini-Mental State Examination (MMSE) Revised Philadelphia Geriatric Center Morale Scale (PGC morale scale) Multidimensional Observation Scale for Elderly Subjects (MOSES) Trail Making Tests (TMT-A and TMT-B) Word Fluency Test (WFT)	Quantitative data collection: self-reported data, demographic and health data, behavioral data, standardized cognitive tools.

Notes: To ensure terminological consistency and given that samples of the included studies primarily consisted of individuals with Mild Cognitive Impairment (MCI) and Dementia, we have chosen to use the more general term patients with cognitive impairment where appropriate. In this study, this umbrella term specifically refers only to individuals diagnosed with MCI or Dementia; N/A = Not Applicable.

**Table A3:** Characteristics of therapeutic designs adopted.

Reference	Hardware and Software Equipment	RT Materials ^a^	RT Material Interactions ^b^	Customization VR Environment
Abdalrahim et al. [17]	HW: - HMD VR headsets (unknown model) SW: - ad hoc developed Arabic-language VR content	- images: LA ^c^ 360-degree photos from locations within Jordan - videos: LA ^c^ 360-degree videos from locations within Jordan - audio: typical music from Jordan Stakeholder input (caregivers, dementia specialists, and local cultural advisors) shaped the VR content, ensuring relevance to participants’ backgrounds and personal histories	- LA ^c^ images and video: patients had the possibility to watch them in 360 degrees and navigate them via ad hoc VR app - audio: music could be run by the patient	collective history (not customized)
Afifi et al. [32]	HW: - Oculus Go VR headsets (3-degree-of-freedom) - tablet (type not specified) SW: - Rendever (platform for shared VR experiences) - OpenPose (open source body recognition software program)	- images: LA ^c^ 2D from personal history - video: LA ^c^ 2D from personal history + LA ^c^ 360-degree videos from Rendever - unknown: “*…In VR Session 1 (Virtual Adventures), the dyad chose five travel adventures (*e.g., *safari, hot air balloon, boat ride in Thailand) among 25 possible preprogrammed adventures…*”	- multiplayer co-presence in the same environment - Selection of a virtual experience/environment (3D CG environment browsing) - 3D LA ^c^ image/video browsing - 2D image/video browsing	personal history (customized) + collective history (customized)
Brimelow et al. [29]	HW: Samsung Gear VR Google and a Samsung Galaxy S7 mobile phone with preloaded 360-degree video content SW: unknown (360 video-player)	- images: not specified type, non-personal - audio: relaxing music, narration audio	- images: unspecified interactivity level by the patient - audio: background music as a companion for the image exploration	not history-related (non-customized)
Coelho et al. [18]	HW: - Samsung Gear VR Google with Samsung S7 smartphone; - Oculus Rift headset - Earphones - GoPro Fusion 360 camera SW: - captured 360° videos edited with GoPro Fusion Studio and Adobe Premiere Pro (to remove distractions)	- images: LA ^c^ photo from 360-degree camera (from locations according to personal preferences of patients; a finite number of locations were chosen to shoot according to majority of preferences from patients’ interviews.)	- LA ^c^ images: patients had the option to watch them in 360 degrees and navigate them via commercial VR app (unspecified)	personal history (customized)
Huang et al. [8]	HW: a VIVE Pro VR HMD SW: ad hoc-developed VR app	- images: 2D photos from personal history; - objects 3D: CG 360 degrees from 3D digital asset - audio: music according to personal preferences, narration	- 2D photos/music: patient had the possibility to watch/play them within a CG room and navigate across them via an ad hoc VR app - 3D objects could be manipulated by patients (e.g., feeding virtual chickens)	personal history (customized) + collective history (not customized)
Saredakis et al. [13]	HW: Meta Oculus Go (VR headset) SW: YouTube VR, Wander (Google Street View)	- images: location-based LA ^c^ 360-degree photos available via Google Street View - video: LA ^c^ 360-degree videos available on YouTubeVR (VR content was tailored for each participants after an interview based on reminiscence therapy guidelines)	- LA ^c^ location-based images: patient had the possibility to watch them in 360 degrees (via Wander app) - LA ^c^ videos: patient had the possibility to watch them in 360 degrees (via YouTubeVR app)	not history-related (customized)
Saredakis et al. [30]	HW: - Meta Quest (VR headset) - laptop used (active control group) SW: - YouTube VR and Wander (Google Street View) - YouTube and Street View (for laptop based experiences)	- images: location-based LA ^c^ 360-degree photos available via Google Street View (both HMD and laptop sessions) - video: LA ^c^ 360-degree videos available on YouTubeVR + 2D for laptop sessions (YouTube) (VR content was tailored for each participants after an interview based on reminiscence therapy guidelines)	- LA ^c^ location-based images: patient had the possibility to watch them in 360 degrees (via Wander app or Google Street View on a laptop) - LA ^c^ videos: patient had the possibility to watch them in 360 degrees (via YouTubeVR app or YouTube on a laptop) (either actively or passively)	not history-related (customized)
Siriaya et al. [19]	HW: - Microsoft Kinect sensor - projector (unspecified type) SW: - 3 VR apps developed with Unity3D and the Zigfu development Kit	Image: 3D CG (old posters, magazines, TVs, books) collective history Audio: music (collective history) Interactions: rowing and jogging	- 3D CG objects manipulation pick (pick-up items on the table by moving their hands) - 3D CG environment navigation with rowing or jogging gesture with upper limbs	collective history (not customized) + not history-related (not customized)
Sun et al. [15]	HW: - Meta Quest 3 VR headsets SW: - familiarization with VR through existing apps on Oculus - ad hoc-developed VR app	- images: 2D photos from personal history - objects 3D: CG images in 360 degrees (natural environments) - videos: 2D videos from personal history - audio: music according to personal preferences	- 2D photos/video/music: patient had the possibility to watch/play them within a CG room and navigate across them via an ad hoc VR app - CG images: patient had the possibility to watch them in 360 degrees and move within them (or interact) through standard Oculus apps	personal history (customized) + not history-related (not customized)
Sun et al. [16]	HW: a television set with both the WBRT and NIRT apps SW: ad hoc-developed - Webapp (WBRT) - Non-immersive VR app (NI-VRRT)	- images: 2D photos from personal history, LA ^c^ 360-degree images of familiar places - videos: 2D videos from personal history, YouTube videos of personal preferences - audio: music according to personal preferences, personal recordings	- 2D photos/images/music: patient had the possibility to watch/play them within a CG room and navigate across them via an ad hoc NI-VR (non-immersive) app - LA ^c^ 360-degree images could be watched from within a CG-generated snowball - 2D videos: patient had the possibility to watch them, control playback, access YouTube - 2D photos of familiar places were used to customize the CG room (view from outside the living room)	personal history (customized) + not history-related (customized)
Tominari et al. [31]	HW: - 9.7” iPads panoramic photos of the same subjects. - conventional printed photos of the same subjects as per iPad	- images: 2D photos and LA ^c^ photos from 360-degree camera captured from a museum	[for iPad Non immersive VR only] - LA ^c^ images: patients had the chance to watch them in 360 degrees using an iPad	collective history (not customized)

Notes ^a^ refers to which kind of reminiscence material has been used. ^b^ indicates how VR content was proposed to users; the focus here is on the kind of interactivity, not on the experimental procedure used. ^c^ LA: live action. Notes: To ensure terminological consistency, and given that samples of the included studies primarily consisted of individuals with mild cognitive impairment (MCI) and dementia, we have chosen to use the more general term “patients with cognitive impairment” where appropriate. In this study, this umbrella term specifically refers only to individuals diagnosed with MCI or dementia.

## Appendix B

**Table A4:** Summary of results from the thematic analysis (Theme 1: Participation and Engagement).

Emergent Subthemes	Description of Subthemes	Frequency	Quote from Literature (Examples from Some of the Papers Included)
**High participation rate**	This theme addresses the importance of the participation of a large percentage of the subjects, improving the representativeness of the data, increasing the validity of the results, and reducing the risk of bias or non-random selection.	10 (90.9%)	*“The participation rate was 85% (17/20); most responses to measures were positive. Access to a wide range of freely available content and the absence of technical difficulties made the delivery of a VR reminiscence intervention highly feasible”* [13]
**Sustained engagement**	This theme refers to the continuous and consistent involvement of individuals in the activity over time, leading to active and lasting participation and contributing to the long-term success and more significant results.	9 (81.8%)	*“Moreover, various indicators, including facial expression, eye contact, physical engagement, verbal tone and expression, exhibited significant improvements, further supporting the positive impact of the intervention”* [17]
**More engagement during I-VRRT vs. NI-VRRT**	This theme refers to the greater involvement and interaction that users experience when using I-VRRT compared to a regular flat screen (NI-VRRT). I-VRRT offers an immersive experience that stimulates more the senses and attention, creating a stronger sense of presence in the VR environment, which leads to active and focused participation, while with a flat screen the experience is more passive and less engaging.	3 (27.3%)	*“Results from the questions asked in the session record found that 73% (11/15) of participants in the VR group preferred viewing content in VR to a flat screen if given a choice”* [30]
**Immersion and active participation**	This theme refers to deep and active involvement in the activity, where the individual is physically present but also emotionally and cognitively engaged, contributing significantly to the experience with a positive impact on engagement and overall outcomes.	6 (54.5%)	*“Immersive VR significantly reduces stimuli from physical reality, leading to a heightened sense of presence and engagement within the virtual environment”; ““The high level of visual realism and 360-degree immersion offered by VR headsets facilitates triggering autobiographical memories, enhancing the overall therapy experience”* [18]
**Enhanced sense of presence**	This theme refers to the amplified feeling of being physically and mentally immersed in the virtual environment, where participants perceive the environment as realistic and engaging, enhancing interaction, and overall experience, and contributing to the validity of the data collected.	5 (45.5%)	*“Spatial Presence was measured on a five-point scale, with one meaning “Not at All” and five meaning “Extremely.” Participants’ median score of 4 meant they were in the virtual world. The Social Presence utilized a Likert Scale with responses ranging from one to six, with one meaning “Strongly Disagree” and six meaning “Strongly Agree.” Participants’ median score of 5 indicates they felt the presence of their partner”* [15]

Notes: To ensure terminological consistency and given that the samples of the included studies primarily consisted of individuals with mild cognitive impairment (MCI) and dementia, we have chosen to use the more general term “patients with cognitive impairment” where appropriate. In this study, this umbrella term specifically refers only to individuals diagnosed with MCI or dementia.

**Table A5:** Summary of results from the thematic analysis (Theme 2: Usability and Feasibility).

Emergent Subthemes	Description of Subthemes	Frequency	Quotes from Literature (Examples from Some of the Papers Included)
**Integration of VR into Care Programs is Feasible**	In a research context, this means that the study has found that using VR as part of the treatment or support for patients is practicable, achievable, and can be adapted to existing treatment protocols. The term “feasible” implies that, after examining various factors (such as the availability of technologies), the implementation of a VR-based therapy seems possible and advantageous.	10 (90.9%)	*“This study found that VR using HMDs to deliver reminiscence therapy is feasible and may be beneficial for aged care residents with apathy (…). “This study provides initial evidence that it is feasible to use VR with HMDs for therapy to treat symptoms of apathy in older adults in residential aged care”* [13]
**Ease of Use**	The VR sessions are designed with specific patient needs in mind, ensuring the technology is accessible, understandable and usable without frustration. The ease of use in this context concerns how easily patients can interact with the technology and how much it facilitates the achievement of therapeutic goals without technological obstacles.	11 (100%)	*“We learnt that a key challenge in designing interaction for people with dementia was in finding a balance between the complexity of the virtual activity and the simplicity of the user interaction. Increasing the level of user interaction provides more rooms for potential engagement, but could also increase the level of complexity and thus alienate less capable residents”* [32]
**User-Friendly Interface**	In the research context, this theme is presented and analyzed to ensure accessibility, enhance user experience, reduce technological stress, and promote experiment adherence.	4 (36.4%)	*“The VRRT interface had hypermedia capabilities that allowed participants to link together different multimedia elements nonlinearly to increase accessibility and lessen the cognitive load”* [17]
**High Satisfaction with Navigation and Controls**	In this theme, researchers aim to understand the level to which the technology is usable both for patients and caregivers in terms of ease of navigation through the VR environment and controls.	4 (36.4%)	*“Participants could use hand controls or fingers to navigate the virtual world. Most participants did not experience challenges using the hand controls, and some stated that it simplified navigating the virtual world”* [15]
**Simplifying Controls to Enhance Accessibility and Usability**	This theme is related to the necessity of having simple and understandable VR technologies to better improve control and accessibility.	3 (27.3%)	*“There were concerns for some PWCI using the hand controls related to remembering the functions of the buttons and their ability to coordinate and operate both hands simultaneously.”* [15]
**Overall Well-Tolerated Tools (Rare/Manageable Simulator Sickness Reported)**	This theme refers to the presence of cases of motion sickness in an overall context where participants’ usage of VR technologies has not brought side effects.	4 (36.4%)	*“No significant adverse effects of the intervention were detected, regarding simulator sickness and psychological and behavioral symptoms. However, there were a few cases of minor increases in eyestrain, fullness of head, anxiety, irritability”* [18]
**Assistance Required for Web App Navigation**	Researchers highlight this theme to enhance the importance of improving assistance during VR sessions, as older patients and patients with cognitive impairment might not have the ability to navigate the web app themselves.	3 (27.3%)	*“Participants were not given access to the controllers, and all navigation was performed by the researcher. This was done to minimize frustration and provide a seamless experience for each participant”* [13]
**Adaptability to Resident Needs**	This theme refers to the need for technologies to be adaptable to the context and the needs of patients living in residences to enhance the possibility of better results and participation in the experiments.	4 (36.4%)	*“The program was open to all residents, where a signed consent form could be obtained. […] There were nine participants with dementia and all participants had some level of cognitive impairment. Four residents had mild cognitive impairment, two had moderate impairment, and seven had severe impairment”* [29]
**Caregiver Acceptance**	Caregiver acceptance is essential in VR reminiscence sessions with elderly patients or those with cognitive impairments, as it facilitates the use of technology, ensures patient safety, enables the assessment of intervention benefits, and encourages patient participation, fostering the sustainability and therapeutic success of the sessions.	1 (9.1%)	*“Overall, family members reported high levels of satisfaction. Mean (overall) ratings of satisfaction (M = 8.56) and interest (M = 8.90) were high. The virtual experiences were rated as fun (M = 8.44) and easy to use (M = 8.28), and participants felt very secure (M = 9.30). Family members reported that they would be highly likely to use VR again if it was available (M = 8.41), and to recommend it to a friend or family member (M = 8.68)”* [32]

Notes: To ensure terminological consistency and given that the samples of the included studies primarily consisted of individuals with mild cognitive impairment (MCI) and dementia, we have chosen to use the more general term “patients with cognitive impairment” where appropriate. In this study, this umbrella term specifically refers only to individuals diagnosed with MCI or dementia.

**Table A6:** Summary of results from the thematic analysis (Theme 3: Emotional and Interpersonal Relationship Impact).

Emergent Subthemes	Description of Subthemes	Frequency	Quotes from Literature (Examples from Some of the Papers Included)
**Positive Emotional Impact**	This theme highlights studies pointing out the importance of positive outcomes derived from VR sessions with patients, enhancing the emotional impact that VR technologies can have on users.	10 (90.9%)	*“(…) during the entirety of the program, most experiences (83%) appeared to be pleasant for the participants (…)”* [18]
**Mood Restoration**	This theme refers to the process of restoring a positive or balanced emotional state through targeted interventions. In the context of virtual reality (VR) sessions, it involves using immersive experiences to alleviate negative emotional states and an overall improvement in psychological well-being. This goal is particularly significant as a positive mood can enhance quality of life and support both cognitive recovery and social interaction.	4 (36.4%)	*“whereas in our study, VR reminiscence significantly reduced depressive symptoms. As an interesting and enjoyable tool, VR intervention may be more effective than traditional reminiscence in improving mood”* [8]
**Reduction in Apathy Scores**	This theme is analyzed to demonstrate the possibility of reducing apathy in patients with high scores using VR as a tool to reactivate patients’ interest and commitment.	4 (36.4%)	*“The experience of VR administered by a researcher and leisure and lifestyle coordinator as a leisure activity, significantly reduced apathy in residents (Z = −2.818, p = 0.005) through observations of increased facial ex- pression, eye contact, physical engagement, verbal tone, and expression”* [29]
**Increased Enjoyment and Likeability**	This theme reflects the importance of the impact of VR technologies in increasing the experience of the users with better enjoyment and likeability due to personalized scenarios.	4 (36.4%)	*“Residents indicated that VR was enjoyable with low levels of physical and emotional discomfort reported or observed”* [29]
**Enhanced Social Presence and Copresence**	This theme analyzes social presence as the feeling of being with another person in a virtual world. It allows people to transcend their location in space and feel as if they are with each other psychologically. A component of social presence is the notion of copresence or “the degree to which the observer believes he/she is not alone and secluded, their level of peripheral or focal awareness of the other, and their sense that the other is peripherally or focally aware of them”.	5 (45.5%)	*“The individualized and strength-based approach of reminiscence therapy, regardless of traditional or VR format, fosters positive emotional experiences and social connections”* [27] *“The Environmental Presence is a Likert Scale similar to the Spatial Presence. It assessed participants’ sense of immersion in the virtual world. The median score of 4 meant very immersed in the virtual world”* [15]
**Strengthened Resident–Caregiver Relationships**	This theme refers to the possibility of creating opportunities for positive interaction and emotional sharing between residents and caregivers. During sessions, caregivers can support residents by facilitating the use of technology and engaging in the VR experience. This active involvement fosters a sense of collaboration, improves communication, and could help build a better connection through shared experiences and moments of enjoyment.	5 (45.5%)	*“With respect to the impact on caregiving, the following were identified: i) Relationship building with PwCI”; “Finally, for the feasibility during the COVID-19 physical distancing, the following were identified: i) Improves social connection, ii) Family involvement in care”* [16]
**Reduction in Anxiety Symptoms**	This theme highlights the use of VR on reducing anxiety by providing an immersive and controlled environment that can distract patients from stressful thoughts, promote relaxation through calm or familiar scenarios, and stimulate positive emotions. Additionally, personalized content can help patients feel safer and relive pleasant experiences, contributing to emotional comfort.	7 (63.6%)	*“The findings demonstrate a statistically significant reduction in anxiety levels following the use of VRRT for PwCI. According to the Hospital Anxiety and Depression Scale (HADS) cut-off score, anxiety is indicated at 10 and above”* [17]
**Emotional Connection and Nostalgia**	Researchers underline how VR reminiscence experience could help patients and users remember past events, which could enhance both a sense of nostalgia due to time passing by and an emotional and social connection among participants and between participants and caregivers. Sharing memories with people can evoke emotions that allegedly create a connection.	5 (45.5%)	*“No participant remained silent during the experience and their discourse often included memories of their personal experiences associated with the locations displayed in the videos (71.1%)”* [18]
**Sense of Identity and Personal Memories**	This theme highlights the importance of VR reminiscence intervention as a source of improving users’ recollection of personal memories and sense of identity, which may be diminished due to cognitive impairments and other health conditions.	4 (36.4%)	*“Furthermore, results suggest that immersion with 360◦videos, specifically of locations that are relevant to each individual’s life story, may lead people with dementia to reminisce and reenact stories from their past, often spontaneously”* [8]

Notes: To ensure terminological consistency and given that the samples of the included studies primarily consisted of individuals with mild cognitive impairment (MCI) and dementia, we have chosen to use the more general term “patients with cognitive impairment” where appropriate. In this study, this umbrella term specifically refers only to individuals diagnosed with MCI or dementia.

**Table A7:** Summary of results from the thematic analysis (Theme 4: Cognitive Functions Outcomes).

Emergent Subthemes	Description of Subthemes	Frequency	Quotes from Literature (Examples from Some of the Papers Included)
**Significant cognitive improvement**	This theme underlines the relevance of VR interventions in the improvement of cognitive functioning in patients with cognitive impairment and higher levels of apathy.	2 (18.2%)	*“These findings suggest noteworthy improvements in cognitive functioning as a result of the intervention”; “These findings suggest noteworthy improvements in cognitive functioning as a result of the intervention”* [17]
**No significant improvement in cognitive function**	This theme demonstrates the relevance of negative or non-positive results expected during research, highlighting the importance of no results in reorienting the experimental methods of the research.	3 (27.3%)	*“The results of this study are inconclusive, with no significant differences found between the intervention groups. Median scores for apathy decreased in the active control group and remained the same in the VR group from baseline to follow-up (Figure 3). Results from the session record for the VR group indicated that there was a preference for viewing content in VR than on a flat screen. […] The enjoyment or preference for VR did not translate to improved outcomes over flat screen technology”* [30]
**Enhanced memory recall**	This theme emerged in the research where VR reminiscence sessions enable patients to reactivate the ability to recall from memory despite mild or major cognitive impairments.	3 (27.3%)	*“(…) similar small effect sizes with higher scores in the active control group were reported for responsiveness and memory”* [30]

Notes: To ensure terminological consistency and given that the samples of the included studies primarily consisted of individuals with mild cognitive impairment (MCI) and dementia, we have chosen to use the more general term “patients with cognitive impairment” where appropriate. In this study, this umbrella term specifically refers only to individuals diagnosed with MCI or dementia.

**Table A8:** Summary of results from the thematic analysis (Theme 5: Challenges).

Emergent Subthemes	Description of Subthemes	Frequency	Quotes from Literature (Examples from Some of the Papers included)
**Physical Comfort of Equipment**	This theme highlights the importance of focusing on the physical comfort of patients undergoing a different type of intervention with technological devices that affect their vision or movement.	9 (81.8%)	*“All participants felt the HMD’s adjustable straps and lightweight increased comfort when wearing it; “They requested (a) adjustable headsets that accommodate glasses and are comfortable for PWCI”* [15]
**Navigation Complexity for Older Users**	Researchers focus on challenges that older users may undergo with using technologies, focusing on how to better improve technology impact and usability.	8 (72.7%)	*“There were concerns for some PWD using the hand controls related to remembering the functions of the buttons and their ability to coordinate and operate both hands simultaneously. “Too complicated, there are too many buttons (…) you are doing it with both hands”(CP#3)”* [15]
**Physical Motion as a Critical Barrier**	This theme is mentioned in several papers as physical motion can impact the user’s well-being with sickness and, more specifically, cybersickness.	7 (63.6%)	*“The participants remained seated during the VR intervention to reduce motion sickness when using the HMD and to also minimize the risk of falls in the elderly”* [8]
**Interactive Features in VR**	This theme focuses on the inclusion of interactive elements in VR experiences, allowing users to actively engage with their environment. Features like object manipulation, decision-making opportunities, and interactive narratives enhance user immersion, foster learning, and improve the overall effectiveness of VR applications.	2 (18.2%)	*“Participants gained familiarity with the broad spectrum of the uses of VR as they experienced the VR environments through virtual travel to different nature scenes and engaged in stimulating activities such as painting in a 3D virtual environment”* [15]
**Limited Interpersonal Interaction**	This theme highlights the potential reduction in real-world interpersonal interactions when using VR. While VR environments can simulate social settings, they may inadvertently isolate users from meaningful face-to-face communication. Addressing this requires a balance between virtual and real-world interaction to ensure users maintain social connections outside the VR experience.	1 (9.1%)	*“Although VR provides increased immersion and realism, the interaction between the participant and researcher or therapist can be compromised by the HMD. Improving outcomes related to neuropsychiatric symptoms or quality of life may be influenced by interactions with the researcher or therapist. For example, reminiscence therapy traditionally involves attentive behaviors, including eye contact and body language [75]. Wearing the HMD during the interventions did not provide the same level of personal interaction as in the active control group. This may explain why VR in this study did not demonstrate the same positive outcomes seen in VR research using exposure and distraction-based therapies [35,36], where there is increased focus on the content being viewed rather than interaction with the therapist”* [29]
**Different Levels of Familiarity with Technology that Impact on the Ability to Adapt to the VR Environment**	This theme acknowledges the varying degrees of technological familiarity among users, which can significantly impact their ability to adapt to and benefit from VR experiences. Solutions include offering clear instructions, user-friendly interfaces, and training sessions to ensure users of all skill levels can comfortably and effectively engage with the VR environment. The availability and accessibility of the necessary technology, such as head-mounted displays (HMDs) and VR software, may pose logistical challenges, especially in under-resourced care homes.	4 (36.4%)	*“Future work will focus on improving the user experience for the caregivers to improve the upload of media, cross-platform compatibility for access through diverse devices, and video tutorials for independent use without requiring technical assistance”* [16]
**Participant Engagement Variability**	Some participants exhibited varying levels of engagement during sessions, requiring session lengths to be adjusted to accommodate individual tolerance and interest levels.	4 (36.4%)	*“Although VR provides increased immersion and realism, the interaction between the participant and researcher or therapist can be compromised by the HMD. Improving outcomes related to neuropsychiatric symptoms or quality of life may be influenced by interactions with the researcher or therapist”* [30]

Notes: To ensure terminological consistency and given that the samples of the included studies primarily consisted of individuals with mild cognitive impairment (MCI) and dementia, we have chosen to use the more general term “patients with cognitive impairment” where appropriate. In this study, this umbrella term specifically refers only to individuals diagnosed with MCI or dementia.

**Table A9:** Summary of results from the thematic analysis (Theme 6: Expressed Preferences related to the VR scenarios).

Emergent Subthemes	Description of Subthemes	Frequency	Quotes from Literature (Examples from Some of the Papers Included)
**Enjoyment for Nature-Based VR Content**	This theme highlights how nature-based VR content could create a more relaxing environment in users, helping to create an enjoyable experience.	2 (18.2%)	*“Scenes preferred by residents included a farmyard scene comprising goats and chickens, an underwater scene of the Great Barrier Reef, a beach scene, and a scene comprising penguins in the snow”* [29]
**Realistic Elements**	Researchers underline the importance of realistic features in VR sessions to better improve memory recall and reminiscence therapy.	7 (63.6%)	*“The color. It seemed so natural to me because I lived down there. It was lovely to see it”* [29]
**Enjoyment for Reminiscence-Oriented Content (like familiar or meaningful locations)**	Familiar or meaningful locations better improve memory recall from users and create a space where they could feel safer and in line with their story.	11 (100%)	*“The most common response about what the participants liked about the experience was related to reminiscence and the enjoyment of seeing places from their past”* [13]
**Variety in Content**	This theme emphasizes the importance of offering diverse content in VR experiences to cater to different user preferences, goals, and needs. A broad range of scenarios, activities, and narratives enhances engagement and allows for tailored experiences that can meet various cognitive, emotional, or therapeutic objectives.	2 (18.2%)	*“videos and audio, to evoke individual and group ‘positive memories”* [17]
**Relaxing Themes**	Relaxing themes are one of the focuses of researchers as they could improve users’ performance in reminiscence therapy, helping to explore an environment based on positivity.	9 (81.8%)	(Outdoor and calming themes, such as mountain landscapes, beaches, and serene farm scenes, were specifically designed to engage and soothe residents): *“The 360-degree videos contained within the library consisted of relaxing scenes, such as underwater themes, beaches, farmyard animals, travel destinations, and snowscapes that were specially created for the aged care industry. These videos lasted between 4 and 5 min each, with the perception changing in response to the viewers head movements. Scenes also consisted of relaxing background music and narration about the relevant scene. (…) Residents were engaged in one VR session in an individual or a small group session while at rest. Sessions were run by the leisure and lifestyle coordinator, to provide a relaxing and engaging leisure activity, with documentation completed by the research coordinator. Attempts were also made to implement VR as a diversion therapy in response to acute disruptive behaviors, distress, or agitation at the time of presenting symptoms”* [29]
**Preference for I-VRRT vs. NI-VRRT**	This theme highlights the preference for fully immersive VR environments compared to traditional flat-screen displays. Immersive VR provides a sense of presence and engagement that flat screens cannot match, enhancing the effectiveness of interventions, education, and entertainment by creating a more realistic and captivating experience.	1 (9.1%)	*“Results from the questions asked in the session record found that 73% (11/15) of participants in the VR group preferred viewing content in VR to a flat screen if given a choice”* [30]
**Flexibility in VR Administration**	This theme addresses the need for adaptable VR administration, allowing for adjustments in session timing, content delivery, and user interaction. Flexibility accommodates users with varying schedules, capabilities, and preferences, ensuring that the technology can be effectively implemented in diverse settings and populations.	6 (54.5%)	*“The length of the session was adjusted to take into account the participant’s level of engagement”* [17]

Notes: To ensure terminological consistency and given that the samples of the included studies primarily consisted of individuals with mild cognitive impairment (MCI) and dementia, we have chosen to use the more general term “patients with cognitive impairment” where appropriate. In this study, this umbrella term specifically refers only to individuals diagnosed with MCI or dementia.

**Table A10:** Summary of results from the thematic analysis (Theme 7: Technical Design Recommendations).

Emergent Subthemes	Description of Subthemes	Frequency	Quotes from Literature (Examples from Some of the Papers Included)
**Adjustments for Better Visual and Audio Feedback**	This theme highlights the importance of refining visual and auditory elements in VR to improve the overall user experience. Enhancements include higher-resolution graphics, immersive 3D soundscapes, and synchronized feedback to create a more engaging and realistic environment.	10 (90.9%)	*“They requested (a) adjustable headsets that accommodate glasses and are comfortable for PWCI”* [15]
**Improved Object and Camera Rendering for Realism**	This focuses on optimizing object modeling and camera rendering to achieve lifelike visuals in VR. Attention to detail in textures, lighting, and object physics is emphasized to ensure a more authentic and immersive experience.	4 (36.4%)	*“To further add immersion, three snow globes on the table beside the window are accepted 360° panoramic image files as attachments (Figure 2c). When attached, the snow globes will display the panoramic image as a miniature environment. During therapy, the user can view the image by moving the camera inside of the snow globe, therefore rotating the view in an immersive viewing experience. Moreover, one exterior element is able to accept a 360° panoramic image file as an attachment to create an immersive outside view* [16]
**Tangible Interfaces for Non-Touch-Based Users**	This theme addresses the need for alternative interaction methods for users who may not be able to utilize standard touch or motion-based controls. Tangible interfaces such as physical props, voice commands, or adaptive devices are proposed to enhance accessibility.	4 (36.4%)	*“For accessibility considerations, there were concerns regarding the usability of the hand controls for PWD and the request to include activities that do not require the use of hand controllers”* [16] *“Tangible interfaces were preferred by participants who found hand controls overly complex, indicating the need for physical objects or simplified interaction methods”* [18]
**Incorporation of Personalized Reminiscence Content**	This emphasizes the inclusion of individualized content in VR experiences, particularly for therapeutic applications. By integrating personal memories, familiar settings, or meaningful imagery, users can engage more deeply, fostering emotional connections and enhancing outcomes.	5 (45.5%)	*“However, each person has a unique history, and using individualized content has been demonstrated to be more effective than using generic content”* [13]
**Enhanced Interactivity**	This theme focuses on increasing the level of interactivity in VR environments, allowing users to actively engage with virtual objects, characters, and scenarios. Enhanced interactivity improves immersion and enables more meaningful user participation.	4 (36.4%)	*“They expressed the desire to engage in more interactive activities, such as exergames, as described by one participant. “Something where you are doing an exercise class. Having to follow directions, […] versus just being immersed without any actions”* [15]
**Improving/Simplifying Navigation**	To make VR accessible to a broader audience, this theme highlights the importance of intuitive and straightforward navigation systems. Simplified controls, clear prompts, and user-friendly interfaces are recommended to minimize frustration and confusion.	3 (27.3%)	*“There were concerns for some PWD using the hand controls related to remembering the functions of the buttons and their ability to coordinate and operate both hands simultaneously. “Too complicated, there are too many buttons [….] you are doing it with both hands” (CP #3). A similar challenge was expressed regarding using fingers to navigate the virtual world, as some PWD experienced difficulties grabbing and picking up items in the virtual world”* [17]
**Minimizing Cybersickness symptoms**	This addresses the discomfort caused by VR, such as nausea or dizziness, often referred to as cybersickness. Strategies include optimizing frame rates, reducing motion inconsistencies, and designing environments that limit rapid or disorienting movements.	2 (18.2%)	*“Participants remained seated during the VR content exposure, as standing has been found to increase the risk of motion sickness when using HMDs”* [13]
**Facilitator Assistance: Provide trained staff to guide participants during sessions for a smoother experience**	This theme underscores the importance of having trained facilitators available during VR sessions to guide participants, troubleshoot issues, and ensure a smoother and more supportive experience, especially for first-time or vulnerable users.	5 (45.5%)	*“Additional feedback from an interview conducted after the completion of the SUS questionnaire provided the suggestion described in the following sections. 1) Web-based RT: The caregivers provided the following suggestions […] Regarding what could make the system easier to use, the feedback indicated the following: i) assistance from someone to help use the system”* [16]
**Scene Selection Flexibility**	This focuses on offering users a variety of scenes or scenarios to choose from during VR sessions. Flexibility in scene selection allows customization to suit individual preferences, needs, or therapeutic goals, enhancing the relevance of the experience.	2 (18.2%)	*“During our sessions, the intervention group using VR could freely enlarge, shrink, and adjust the angles of their panoramic images”* [31]
**Improve the Comfort and Fit of Headset Devices**	Recognizing the physical discomfort that can result from poorly fitting or heavy VR headsets, this theme advocates for ergonomic designs that provide better weight distribution, adjustable straps, and comfortable materials to accommodate extended use.	6 (54.5%)	*“One participant reported that the HMD was heavy; however, this was rectified by adjusting the head strap”* [13] *“They requested (a) adjustable headsets that accommodate glasses and are comfortable for PWCI”* [15]
**Recommendation for Short VR Session to Prevent Fatigue and Overstimulation**	This theme emphasizes limiting session duration to prevent user fatigue, overstimulation, or discomfort. Shorter, focused sessions are recommended, particularly for first-time users or those with specific health concerns, ensuring a positive experience without overburdening participants.	3 (27.3%)	*“The VRRT intervention comprising 10 tailored RT sessions held over the course of five weeks…” indicates structured and relatively brief sessions to optimize benefits and minimize fatigue”* [17]
**Integration of Hypermedia Features**	Hypermedia capabilities that enable nonlinear navigation between multimedia elements (e.g., videos, images, sounds) are recommended to provide flexibility and reduce the cognitive load.	6 (54.5%)	*“iii) uploading multiple media can help streamline the process as individual uploads take time”* [16] *“The VRRT interface had hypermedia capabilities that allowed participants to link together different multimedia elements nonlinearly to increase accessibility and lessen the cognitive load”* [17]
**Accessibility Features**	VR systems should include adjustable settings for session duration, audio levels, and visual presentation to accommodate physical and sensory limitations.	4 (36.4%)	*(For accessibility considerations, there were concerns regarding the usability of the hand controls for PwCI and the request to include activities that do not require the use of hand controllers): “They requested […] (e) activities that do not require hand controls”* [15]

Notes: To ensure terminological consistency and given that the samples of the included studies primarily consisted of individuals with mild cognitive impairment (MCI) and dementia, we have chosen to use the more general term “patients with cognitive impairment” where appropriate. In this study, this umbrella term specifically refers only to individuals diagnosed with MCI or dementia.

**Table A11:** Summary of results from the thematic analysis (Theme 8: Limitations and Recommendations for Future Studies).

Emergent Subthemes	Description of Subthemes	Frequency	Quotes from Literature (Examples from Some of the Papers Included)
**Lack of Long-Term Cognitive and Behavioral Change**	This theme highlights the challenges in achieving sustainable improvements in cognitive and behavioral domains through virtual reality (VR) interventions. It emphasizes the need for strategies to ensure that positive changes observed during VR sessions extend beyond the intervention period and translate into long-term benefits for users.	4 (36.4%)	*“Previous research has extensively investigated the efficacy of reminiscence therapy in addressing cognitive decline and apathy; however, effects often did not persist beyond the intervention period”* [17]
**Addressing Excessive Motion in VR Experiences**	This focuses on mitigating the challenges associated with excessive motion in VR environments, which can cause discomfort or disorientation. Strategies include designing environments with reduced motion sickness risk and optimizing motion control for user comfort and accessibility.	1 (9.1%)	*“Six participants reported side effects, 4 of which were symptoms related to items measured by the SSQ, including nausea, eyestrain, and dizziness”* [13]
**Ensuring Realism and Engagement in VR Design**	To maximize the effectiveness of VR, this theme focuses on creating realistic and engaging virtual environments that resonate with users, maintaining a balance between immersivity and user comfort to improve emotional and cognitive engagement.	3 (27.3%)	*“Indeed, 360◦ videos currently do not allow to freely change the viewpoint or to interact with the environment. However, they are easier to create—due to the increasing accessibility of 360◦ cameras—and they depict real recognizable settings, which can be vital for reminiscence therapy, as higher realism of VR stimuli can facilitate the recollection of autobiographical memories”* [18]
**Lack of Control Group**	This highlights methodological limitations in VR studies due to the absence of a control group, which undermines the ability to attribute observed outcomes directly to the intervention. Addressing this involves improving study designs to include appropriate comparisons.	4 (36.4%)	*“There was no control group or a group receiving traditional reminiscence therapy; therefore, the results of this study must be carefully interpreted”; “Studies using a control group and comparing the use of VR with traditional forms of reminiscence should be conducted in the future to confirm and develop these findings”* [8]
**Selection Biases**	This addresses the potential skew in results caused by non-representative participant samples, which may limit the generalizability of the findings. Mitigation strategies include inclusive recruitment practices and diverse sample selection.	4 (36.4%)	*“Participant selection for recruitment was performed by staff at the aged care facility. This may have resulted in selection bias within the sample”* [30]
**Adding VR Sessions (multi-session interventions)**	This theme emphasizes the importance of extending VR interventions over multiple sessions to reinforce learning, improve skill retention, and achieve more substantial cognitive or behavioral improvements over time.	10 (90.9%)	*“Studies suggest that reminiscence therapy of more than 8 sessions might be required to obtain therapeutic effects”* [8]
**Limitation of Self-Reported Measures**	This acknowledges the over-reliance on self-reported data, which can be subjective and prone to biases. It suggests the incorporation of objective metrics or third-party evaluations to complement self-reported measures.	4 (36.4%)	*“As the self-rated version of the AES was used, it needs to be considered that people with apathy will normally underestimate their condition. Therefore, true scores may be higher than reported”* [13] *“It is important not to discount the possibility that immediate effects provide enjoyment and stimulation during an intervention for participants that may not translate to longer-term measures”* [30]
**Improving Interaction to Facilitate Navigation of the VRs Themselves**	This theme focuses on enhancing user interaction mechanisms in VR to make navigation intuitive and accessible, reducing barriers for users unfamiliar with VR technology or those with physical limitations.	2 (18.2%)	*“The feasibility of controller usage and navigation in VR in this population is an avenue for future research for studies conducted over a longer period”* [13]
**Improving Sample Size**	This addresses the issue of small sample sizes in VR studies, which reduce the statistical power and reliability of the findings. Increasing sample sizes improves the robustness and generalizability of the results.	9 (81.8%)	*“However, the small sample sizes in both studies may have impacted the statistical significance of results”* [29] *“The study included participants with different conditions who were taking a range of medications, and the sample size was relatively small; these factors affected generalizability and was also a possible reason for the lack of significant differences in outcome measures over time”* [30] *“Studies with larger sample sizes should be conducted in the future to validate our findings”* [8] *“The small sample size; absence of control groups; short intervention program; inclusion of participants that did not attend all intervention sessions”* [18] *“Because this is a feasibility study, the sample size is rather small. A larger sample would have allowed for more comparisons, and potentially more power to find significant differences, between older adults with MCI and dementia”* [32]
**Promote Staff Availability to Facilitate Sessions and Time Constraints that Make it Difficult to Implement VR during Acute Behavioral Episodes**	Recognizing that staff shortages can hinder the effective implementation of VR interventions, this theme calls for strategies to ensure adequate staffing and scheduling flexibility, especially during acute behavioral episodes when VR might be most beneficial.	3 (27.3%)	*“Attempts were made to implement VR as a diversion therapy during acute disruptive behaviors, distress, or agitation (…) This was facilitated by two registered nurses (RNs) within the facility and proved unsuccessful because of practical limitations such as staffing and time delays, and resident factors such as agitation”. “Highlights the challenges in staffing availability for timely interventions”* [29]
**Limited Baseline Equality**	This theme identifies the challenge of ensuring participants in VR studies have comparable baseline characteristics, as discrepancies can affect the validity of the results. Improved randomization and stratification methods are proposed solutions.	3 (27.3%)	*“There was a significant difference between the groups at baseline, which may have also contributed to the lack of statistical differences”* [30] *“Baseline measures were not uniform across all care facilities, adding variability to the study design”* [17] *“Baseline demographic characteristics, cognitive function, and global status of the participants showed variations, which might affect the generalizability of the results”* [8]
**Explore Alternative Interaction Methods**	Study the feasibility of using simpler interaction methods, such as voice commands or hand-tracking, to reduce the cognitive and physical demands of navigating VR environments.	3 (27.3%)	*“Participants provided insightful feedback to ensure that the VR reminiscence therapy addressed their needs. There were concerns regarding the usability of the hand controls for PWCI and the request to include activities that do not require the use of hand controllers.” Suggests exploring non-controller-based interaction methods”* [15]
**Allow Accessibility without Internet**	This theme underlines the need for VR systems that function effectively without requiring constant internet access, increasing usability in settings with limited connectivity and reducing barriers for underserved populations.	1 (9.1%)	*“Even though the WiFi was tested beforehand with Rendever, one of the family members’ internet connections was weak, which disrupted the flow of the VR experience”; “VR technologies designed for older adults often encounter barriers, but creating systems that can operate without dependency on live internet connections ensures broader accessibility”* [32]

Notes: To ensure terminological consistency and given that the samples of the included studies primarily consisted of individuals with mild cognitive impairment (MCI) and dementia, we have chosen to use the more general term “patients with cognitive impairment” where appropriate. In this study, this umbrella term specifically refers only to individuals diagnosed with MCI or dementia.
